# CTCF binding landscape in jawless fish with reference to Hox cluster evolution

**DOI:** 10.1038/s41598-017-04506-x

**Published:** 2017-07-10

**Authors:** Mitsutaka Kadota, Yuichiro Hara, Kaori Tanaka, Wataru Takagi, Chiharu Tanegashima, Osamu Nishimura, Shigehiro Kuraku

**Affiliations:** 1 Phyloinformatics Unit, RIKEN Center for Life Science Technologies, 2-2-3 Minatojima-minami, Chuo-ku, Kobe, 650-0047 Japan; 2 Evolutionary Morphology Laboratory, RIKEN, 2-2-3 Minatojima-minami, Chuo-ku, Kobe, 650-0047 Japan

## Abstract

The nuclear protein CCCTC-binding factor (CTCF) contributes as an insulator to chromatin organization in animal genomes. Currently, our knowledge of its binding property is confined mainly to mammals. In this study, we identified *CTCF* homologs in extant jawless fishes and performed ChIP-seq for the CTCF protein in the Arctic lamprey. Our phylogenetic analysis suggests that the lamprey lineage experienced gene duplication that gave rise to its unique paralog, designated *CTCF2*, which is independent from the previously recognized duplication between *CTCF* and *CTCFL*. The ChIP-seq analysis detected comparable numbers of CTCF binding sites between lamprey, chicken, and human, and revealed that the lamprey CTCF protein binds to the two-part motif, consisting of core and upstream motifs previously reported for mammals. These findings suggest that this mode of CTCF binding was established in the last common ancestor of extant vertebrates (more than 500 million years ago). We analyzed CTCF binding inside Hox clusters, which revealed a reinforcement of CTCF binding in the region spanning Hox1-4 genes that is unique to lamprey. Our study provides not only biological insights into the antiquity of CTCF-based epigenomic regulation known in mammals but also a technical basis for comparative epigenomic studies encompassing the whole taxon Vertebrata.

## Introduction

The CCCTC-binding factor (CTCF), containing the C2H2 Zn finger-type DNA binding domains, plays a pivotal role in chromatin organization as an insulator^1, 2^. Recent studies have revealed that CTCF binds to two-part motifs consisting of core and upstream motifs^3, 4^, and that the deposition of CTCF binding sites topologically determines chromatin structure^2, 5–7^. In mammalians, it was shown that CTCF binding sites propagated through retrotransposition^3, 8^. So far, our knowledge on molecular functions of CTCF is confined mostly to mammalians, whereas *CTCF* orthologs have been identified in diverse bilaterian animals^9^. Remarkably, *CTCF* orthologs are not retained in some bilaterians, which were also revealed to lack clusters of Hox genes^9, 10^. Studies on mammals have elucidated the involvement of CTCF binding in the regulation of chromatin topology in Hox clusters^2, 11, 12^. These findings pinpoint the importance of understanding the mechanism of Hox gene regulation from an epigenomic viewpoint.

Lampreys, used in this study, belong to Cyclostomata, a taxon that diverged from the future gnathostome (jawed vertebrate) lineage more than 500 million years ago, and are characterized by the lack of the articulating jaw^13^. Whole genome sequencing and analysis was first carried out for the sea lamprey *Petromyzon marinus* and later for the Arctic lamprey *Lethenteron camtschaticum* (or Japanese lamprey *L. japonicum*)^14, 15^. These genomic studies revealed its unique features, such as programmed genomic rearrangement and peculiar characters of protein-coding sequences associated with highly biased base composition towards increased GC-content^14, 16^. As these features could be influenced by epigenomic regulation through modifications of chromatin or DNA, it is crucial to investigate epigenomic regulation in this animal group.

In this study we identified two lamprey *CTCF* homologs, including *CTCF2*, which was duplicated in the lamprey lineage independently from the other gene duplication that gave rise to *CTCFL* (also called Boris^17^). We performed, to our knowledge, the first ever analysis with chromatin immunoprecipitation and sequencing (ChIP-seq) for a jawless fish, enabled by antibody validation and ChIP protocol optimization. Our results indicate the establishment of the binding property of CTCF, mediated by the two-part motif, in the last common ancestor of cyclostomes and gnathostomes, and also detected a transposon-associated propagation of the CTCF binding site in the lamprey, as in mammals. Focusing on Hox clusters, we also compared CTCF binding patterns between lamprey and gnathostomes.

## Results

### Generating Gene Models for *L. camtschaticum*

In order to allow comprehensive gene search and related gene-based analyses, genome-wide prediction of protein-coding genes was performed for the *L. camtschaticum* genome assembly LetJap1.0, for which no established gene model was available. The program AUGUSTUS^18^ was trained to adapt to this species using conserved genes and was employed for inference of protein-coding exons by incorporating RNA-seq reads (Supplementary Table S1) of embryonic and tissue samples (as evidence of exons) and protein sequences of other species (as evidence of coding regions). The resultant set of 34,320 predicted genes was further refined to recover false-negative exons and genes and to bridge split exons to retrieve longer open reading frames. This resulted in the final set of 34,435 genes, designated as the gene model ‘GRAS-LJ’ (Supplementary Fig. S1 and Supplementary Table S2; also see Supplementary Materials and Methods for the detailed procedures).

### Identification of Cyclostome CTCF Genes

A BLASTP search was performed to identify CTCF homologs in our gene model, using the human CTCF peptide sequence (NCBI NP_006556.1) as a query. This identified a component of GRAS-LJ, g14920.t1 (3621 bp) that is harbored by the scaffold KE994200.1 of the genome assembly LetJap1.0. The sequence was extended by using RNA-seq data to obtain the transcript sequence of 5781 bp that encompasses the putative full-length open reading frame (ORF), which is markedly longer [1207 amino acids (aa)] than that of already reported orthologs [human, 727 aa (NCBI﻿ NP_0065561); fruitfly, 818 aa (NCBI NP_648109)]. The gene from which this cDNA was derived was designated *LjCTCF* (NCBI KX830966).

Unexpectedly, our search for sequences similar to CTCF identified another one that was different from *LjCTCF*. This was based on the components of GRAS-LJ g14288.t1 and g23728.t1, predicted on the scaffolds KE994121.1 and APJL01108776.1 respectively, and the sequence was extended to 1475 bp with our RNA-seq data and 3′ RACE to cover its putative full-length ORF (396 aa). The gene from which this sequence was derived was designated *LjCTCF2* (NCBI KX830967).

**Figure 1:**
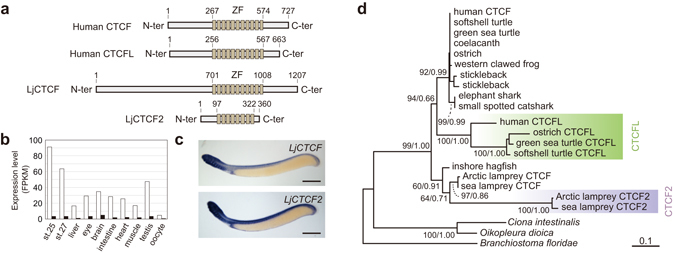
Properties of *L. camtschaticum CTCF* and *CTCF2* genes. (**a**) Protein domain structures, in comparison with human CTCF and CTCFL. The Zn finger domains (ZF) were identified by MOTIF Search (http://www.genome.jp/tools/motif/). See SI Materials and Methods for detail procedures of *LjCTCF* and *LjCTCF2* sequence determination by cDNA cloning. (**b**) Expression levels of *LjCTCF* (white) and *LjCTCF2* (black) in embryos and adult tissues. FPKM, fragments per kilobase of exon per million mapped sequence reads. (**c**) Whole-mount *in situ* hybridization on stage 26.5 embryos. Riboprobes were designed in non-conserved regions downstream to the Zn finger domains to avoid cross-hybridization (for the magnified view and the result with upstream riboprobes, see Supplementary Fig. S10). Note that the difference in the intensities of the expression signals between the two genes do not correspond to that of actual expression levels, as indicated by our RNA-seq data in (**b**). Scale bars: 500 μm. (**d**) Molecular phylogenetic tree. This tree was inferred with the maximum-likelihood approach using 208 aligned amino acid sites (see Supplementary Tables S8 and S9 for the list of sequences used). Two stickleback sequences are included (upper, Ensembl ENSGACP00000003270; lower, ENSGACP00000020939). At each branch node in the tree, only bootstrap value of no less than 60, and the posterior probability inferred with the Bayesian approaches are shown.

The identified *LjCTCF* and *LjCTCF2* sequences were used to further identify their orthologs in sea lamprey (*P. marinus*) and inshore hagfish (*Eptatretus burgeri*). The deduced amino acid sequences of *LjCTCF* and *LjCTCF2* contain eleven and eight Zn finger domains, respectively. Multiple sequence alignment revealed that the elongated stretch upstream to the N-terminus accounts for the large length of LjCTCF (Fig. 1a), and that similarity was not observed between the lamprey CTCF and CTCF2 sequences outside the Zn finger domains (Supplementary Fig. S2).

### Expression Patterns of Lamprey *CTCF* and *CTCF2*

Expression levels of *LjCTCF* and *LjCTCF2* were quantified employing the aforementioned RNA-seq data and our gene model ‘GRAS-LJ’. Both *LjCTCF* and *LjCTCF2* were expressed in all the tissues analyzed, while *LjCTCF* was expressed at a higher level (Fig. 1b). Their detailed embryonic expression patterns were analyzed with whole-mount *in situ* hybridization. This resulted in broad expression signals mainly in the craniofacial regions and axial structures, which is shared between the two genes (Fig. 1c). This pattern is similar to that documented for the CTCF ortholog of zebrafish^19^, clawed frog^20^, and mouse^21^ in that CTCF transcripts were widely distributed, particularly in the brain, branchial arches, and axial structure including the neural tube.

### Timings of *CTCF*-*CTCF2* and *CTCF*-*CTCFL* Duplications

The identification of the second lamprey homolog of *CTCF*, *CTCF2*, prompted us to analyze its evolutionary origin. Our phylogenetic analyses indicated that the duplication between *CTCF* and *CTCF2* occurred in the lamprey lineage, after the split of the hagfish lineage (Fig. 1d). Also, the long terminal branches of *CTCF2* indicated its elevated evolutionary rate. Prior to concluding that *CTCF2* is a lamprey-specific paralog, we sought to carefully examine the possibility that *CTCFL* and *CTCF2* were orthologs, by means of an in-depth analysis in which the likelihoods of all possible tree topologies are computed and statistically evaluated. This analysis did not support the exclusive phylogenetic clustering of *CTCFL* with *CTCF2* (Table 1).

**Table 1:** Tree topology test for the phylogenetic relationship between *CTCFL* and *CTCF2*.

Rank by log-likelihood (lnL)	Tree topology^*^	ΔlnL	*p*AU^†^	*p*KH^‡^
1	((CTCFL,Gn),((La-CTCF2,La),Hf),OG);	ML	0.906	0.726
2	((Gn,((La,La-CTCF2),Hf)),CTCFL,OG);	0.9	0.501	0.274
28	((Gn,(CTCFL,La-CTCF2)),(La,Hf),OG);	10.3	1.00 × 10^−5^	0.034
29	(Gn,((CTCFL,La-CTCF2),(La,Hf)),OG);	10.3	5.00 × 10^−6^	0.034
31	((Gn,(La,Hf)),(CTCFL,La-CTCF2),OG);	10.3	3.00 × 10^−6^	0.034
39^§^	(((Gn,(CTCFL,La-CTCF2)),Hf),La,OG);	15.6	0.024	0.035

^*^Gn, gnathostome CTCF; La, lamprey CTCF; Hf, hagfish CTCF; OG, outgroup CTCF.﻿

^†^ *p*-value of the approximately unbiased test^47^

^‡^ *p* -value of the Kishino-Hasegawa test^48^

^§^The phylogenetic tree supporting the *CTCFL*-*CTCF2* monophyly with the largest *p*AU among the all tree topologies examined.

Abbreviation: ML, maximum likelihood tree.

Previously, *CTCFL* was suggested to have duplicated in the lineage leading to amniotes, by an analysis employing nucleotide sequences without multiple substitutions taken into account^22^. To scrutinize the origin of *CTCFL* more closely, we used amino acid sequences from all major vertebrate lineages, including cyclostomes and chondrichthyans. Our molecular phylogenetic analysis suggested that the gene duplication giving rise to *CTCFL* occurred earlier in vertebrate evolution than previously suggested, that is, before the split of the chondrichthyan lineage (Fig. 1d and Table 2). We further examined if this early duplication timing was supported with variable tree inference methods and sequence datasets. Almost all of the phylogenetic trees inferred exhibited the duplication between *CTCF* and *CTCFL* before the split of the chondrichthyan lineage, while no trees supported the timing of this duplication in the amniote ancestor (Supplementary Fig. S3).

**Table 2:** Tree topology test for the duplication timing between *CTCF* and *CTCFL*

Rank by log-likelihood (lnL)	Tree topology^*^	ΔlnL	*p*AU	*p*KH
1	(((((Amn,Sa),Amp),Te),Ch),CTCFL,Cy);	ML	0.924	0.499
2	((((Amn,(Sa,Amp)),Te),Ch),CTCFL,Cy);	0	0.925	0.501
3	(((((Amn,Amp),Sa),Te),Ch),CTCFL,Cy);	0	0.925	0.501
4	((((Amn,Amp),Sa),(Te,Ch)),CTCFL,Cy);	1.6	0.667	0.22
5	((((Amn,Sa),Amp),(Te,Ch)),CTCFL,Cy);	1.6	0.531	0.22
6	(((Amn,(Amp,Sa)),(Te,Ch)),CTCFL,Cy);	1.6	0.527	0.22
7	(((((Amn,Amp),Sa),Ch),Te),CTCFL,Cy);	1.6	0.376	0.212
8	((((Amn,(Amp,Sa)),Ch),Te),CTCFL,Cy);	1.6	0.378	0.212
9	(((((Amn,Sa),Amp),Ch),Te),CTCFL,Cy);	1.6	0.375	0.212
10	(((Amn,(Sa,(Ch,Te))),Amp),CTCFL,Cy);	1.9	0.55	0.267
106	(((((Amn,Amp),Sa),Te),CTCFL),Ch,Cy);	6.7	0.513	0.139
111	(((((Amn,Amp),Sa),CTCFL),Te),Ch,Cy);	7.3	0.496	0.134
122^†^	((((Amn,CTCFL),(Amp,Sa)),Te),Ch,Cy);	7.9	0.299	0.124
130	(((((Amn,CTCFL),Amp),Sa),Te),Ch,Cy);	8.8	0.02	0.095
139	(((((Amn,Amp),CTCFL),Sa),Te),Ch,Cy);	9.1	0.028	0.091
327^‡^	(((Amn,Amp),CTCFL),(Te,Ch),(Sa,Cy));	12.2	0.069	0.046

^*^Amn, amniote CTCF; Amp, amphibian CTCF; Sa, sarcopterygian CTCF; Te, teleost CTCF; Ch, chondrichthyan CTCF; Cy, cyclostome CTCF.

^†^The phylogenetic tree supporting an amniote CTCF-CTCFL monophyly with the highest *p*AU.

^‡^The phylogenetic tree supporting a tetrapod CTCF-CTCFL monophyly with the highest *p*AU.Abbreviation: ML, maximum likelihood tree.

### Immunodetection of Lamprey CTCF Protein

**Figure 2:**
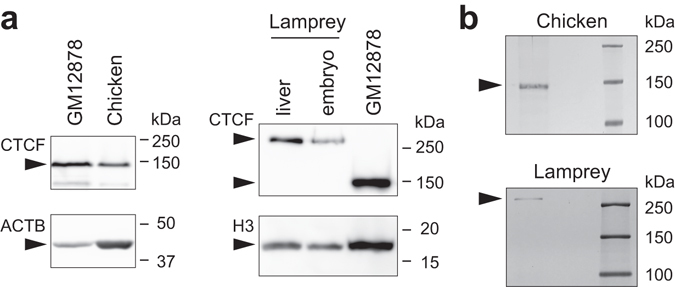
Identification of CTCF proteins. (**a**) Western blotting of CTCF proteins, using an anti-CTCF antibody. Protein extracts from human GM12878 cells, stage 32 chicken embryo, adult lamprey liver, and stage 27 lamprey embryos were used for the analysis. β-actin (ACTB) or histone H3 was used as a loading control protein. (**b**) Immunoprecipitation. Silver-stained SDS PAGE gel of IP proteins showing chicken CTCF protein of approximately 140 kDa, and lamprey CTCF protein of >250 kDa. Detailed procedures of western blotting and immunoprecipitation are described in Supplementary Materials and Methods. Note that the band positions may not be accurate possibly because of posttranslational modification.

For ChIP-seq, we chose a commercially available anti-CTCF antibody that targets the C-terminus region (CST #3418S). Using this antibody, we performed western blotting against protein lysates of human GM12878 cells, a chicken embryo, and the adult liver and embryos of Arctic lamprey. This experiment identified positive bands of approximately 140 kDa for the human and chicken samples, while the band for lamprey was detected at over 250 kDa (Fig. 2a). The increased molecular weight of lamprey LjCTCF protein was consistent with its elongated amino acid sequence mentioned above (Fig. 1a).

Immunoprecipitation was performed to examine whether the antibody specifically recognizes CTCF proteins. Silver staining after SDS-PAGE yielded clear single bands at around 140 kDa for chicken and over 250 kDa for lamprey (Fig. 2b). The gel bands were excised and analyzed with mass spectrometry, which resulted in identification of chicken CTCF and Arctic lamprey CTCF (Supplementary Table S3).

### Genome-wide Detection of CTCF Binding Sites

**Figure 3:**
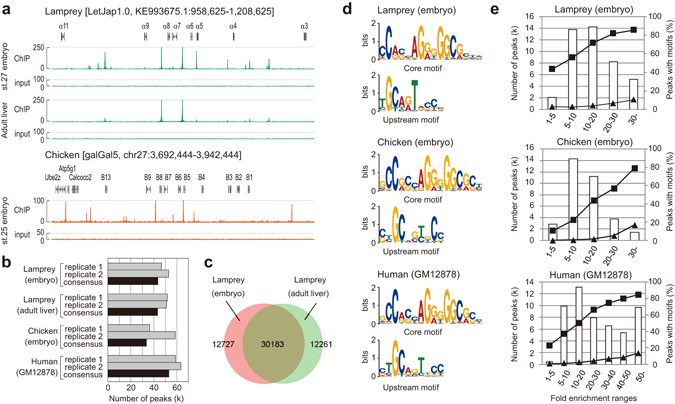
ChIP-seq peaks and binding motifs in lamprey, chicken and human cells. (**a**) ChIP-seq results for lamprey and chicken CTCF. Shown are the lamprey Hox α cluster and chicken Hox B cluster. (**b**) Numbers of peaks identified by MACS2. The “consensus peaks” (black bar) were identified by taking an intersect of peaks called in each replicate and in the merged replicates (see Materials and Methods for details about peak selection). (**c**) Overlap of consensus peaks in the lamprey embryo and adult liver tissue. (**d**) CTCF core and upstream motifs identified by MEME. The motifs were identified in two parts and are almost identical between the different species analyzed (also see Supplementary Fig. S11). (**e**) Peaks in various fold enrichment ranges harboring the core motif and core + upstream motifs. Numbers of ChIP-seq peaks are shown with white bars. Proportions of peaks containing a core motif (▪) and those containing a core + upstream motif (▴) are indicated as lines.

Using the validated anti-CTCF antibody, ChIP assays were performed on Arctic lamprey, chicken, and human samples. Enrichment in the ChIP assays was confirmed by quantitative polymerase chain reaction (qPCR) (Supplementary Fig. S4). ChIP-seq yielded 16–23 million 80nt-long single reads per library, which were mapped to the genomes of the individual species (see Fig. 3a for lamprey and chicken). Proportions of mapped reads were 72–73% for lamprey, 90% for chicken, and 95% for human (Supplementary Table S4). The number of peaks called by MACS2^23^ did not markedly differ between the lamprey liver and embryos (Fig. 3b), and more than 70% of the peaks were shared between the data derived from the liver and embryos (Fig. 3c). This pattern that CTCF binding landscape is intrinsic to individual genomes irrespective of cell types was previously suggested for human^24, 25^. Numbers of significant peaks were comparable among the analyzed species (Fig. 3b and Supplementary Table S5). Nucleotide sequences of core motifs and upstream motifs identified *de novo* by the MEME program^26^, were highly similar among the species (Fig. 3d) and identical to the motifs known for mammals^3, 4, 24^. The validity of the identified binding sites was confirmed with the observation that ChIP-seq peaks with higher fold enrichment more frequently harbor the CTCF binding motif sequence (Fig. 3e).

### Association of CTCF Peaks with Repeats

For mammals, it was shown that CTCF binding sites propagated through retrotransposition, and that the types of retrotransposons that contributed to this process differ even between mammalian taxa^3, 8^. We examined how CTCF binding sites propagated in the Arctic lamprey genome. To this end, a *de novo* repeat sequence library was constructed and used for identification of genomic locations of repetitive sequences (see Materials and Methods for details). We assessed possible overlap between ChIP-seq peaks and the repetitive sequences identified above with Fisher’s exact test. This revealed statistically significant positional association of LTR retrotransposons and DNA hAT transposons with CTCF ChIP-seq peaks (*q* < e-10, odds ratio > 10; Table 3). Particularly remarkable among these was the repetitive sequence #274 in the ‘hAT-Tip100’ repeat subclass, because as many as 80% of the repetitive sequences categorized into this group overlapped ChIP-seq peaks (Table 3). These peaks harbored canonical CTCF binding motifs at a high proportion, without CpG sites for potential DNA methylation, and tended to exhibit high fold enrichment in ChIP-seq, which was validated with qPCR (Supplementary Fig. S5). We queried the hAT-Tip100 sequence in search of counterparts in the genome of a closely related species, *Petromyzon marinus*, which did not yield any high-similarity hit—the most similar sequence showed an E-value of 0.25 for a 28 bp-long nucleotide sequence stretch. This suggests that the propagation of this repetitive element occurred after the split of these two lamprey species, estimated at 30–10 million years ago^27^.

**Table 3:** Association of CTCF binding with repetitive elements.

Repeat ID^*^	Repeat class/subclass^*^	Number of region			Odds ratio	*q*-value^¶^
		Whole genome^†^	ChIP-seq peak associated^‡^	Control^§^		
743	LTR/Gypsy	166	96	2	58.04	1.87E-29
274	DNA/hAT-Tip100	586	489	12	49.73	9.83E-147
771	DNA/hAT-Charlie	145	59	2	35.69	2.47E-17
738	DNA/hAT-Charlie	187	92	4	27.82	7.92E-26
697	LTR/Gypsy	359	137	7	23.70	6.85E-37
239	Unknown	1509	470	26	22.06	2.26E-124
749	DNA/hAT-Charlie	198	45	3	18.13	8.70E-12
828	SINE/tRNA-V	602	59	4	17.83	2.65E-15
337	Unknown	1553	345	24	17.50	9.98E-87

^*^See Materials and Methods, for repeat identification and (sub)class assignment.

^†^Total number of individual repeat regions in the whole genome identified by RepeatMasker.

^‡^Number of repeat regions overlapping with ChIP-seq peaks (summit ± 50 bp).

^§^Number of repeat regions overlapping with control regions (50,000 regions of 100 bp each).

^¶^ *p*-values from Fisher’s exact test subjected to multiple correction by the Q-value package in R.

### CTCF Binding in Hox Clusters

We analyzed distributions of repetitive sequences and CTCF binding sites in Hox gene clusters. First, as performed above for lamprey, repetitive sequences were identified in the whole genomes of chicken, mouse, and human (see Materials and Methods for details). This comparison, based on a uniform condition, revealed a higher extent of repeat intrusion into lamprey Hox gene clusters (37.6% versus 0.5–10.9% in the other species; Supplementary Table S6). The composition of repetitive sequences in the lamprey Hox clusters highly resembled that of its whole genome.

**Figure 4:**
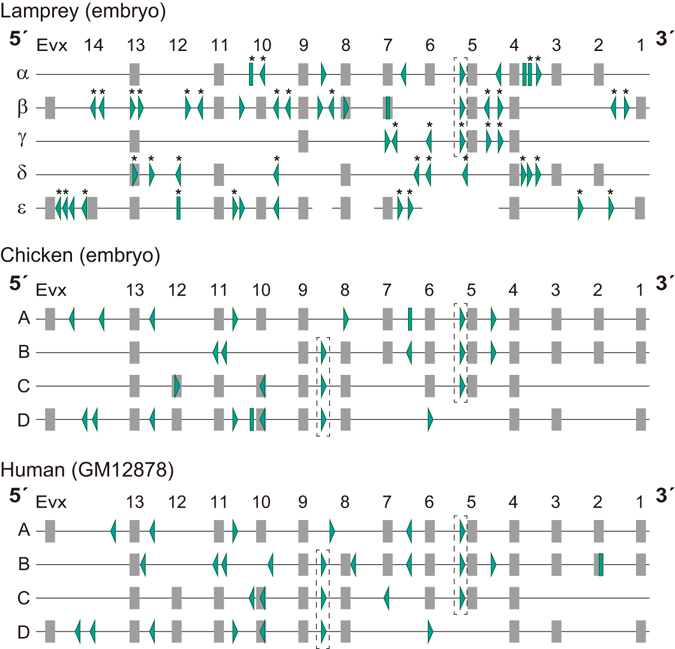
Distribution of CTCF binding sites in Hox clusters. Coding region of genes are indicated with gray boxes. CTCF binding sites are indicated with green arrowheads and bars. Arrowheads indicate the orientations of core motifs inferred by the FIMO program. Green bars indicate CTCF binding sites without a core motif. CTCF binding sites of the Arctic lamprey that overlap repeats are indicated with asterisks (see Results for details). Arrowheads in dashed boxes represent shared relative positions of CTCF binding sites between multiple Hox clusters. This figure includes only “significant peaks” defined in Materials and Methods. See Supplementary Fig. S6 for an equivalent scheme in mouse, dog, and opossum, and Supplementary Fig. S7 for detailed locations of all the peaks in Hox clusters. For the Arctic lamprey, we analyzed only Hox α-ε clusters that were identified in continuous sequences harboring multiple Hox genes.

To perform a cross-species comparison of CTCF binding patterns, we focused on peaks with high fold enrichment (“significant peaks”; see Materials and Methods) and scanned them for binding motifs with their orientations (Fig. 4). The comparison, involving mouse, dog, and opossum for which CTCF ChIP-seq data was publicly available (Supplementary Figs S6 and S7), revealed a high similarity of CTCF binding pattern in the Hox clusters among the gnathostomes analyzed (Fig. 4). The gnathostome commonalities include 1) outward binding motifs located near Evx or Hox13 as well as near Hox5 and 2) shared positioning of binding motifs between the clusters (e.g. between Hox8 and -9 genes, and between Hox5 and -6 genes). In lamprey, we detected more CTCF binding sites (up to 18 sites in lamprey, compared to up to 10 in other species, per cluster), and the outward binding motifs, especially those in the 3′ regions, were located closer to the end of the clusters than in gnathostomes. More conspicuously, we identified multiple CTCF binding sites between Hox1 and -4, in four out of the five clusters covering this segment, whereas no or few peaks were detected in this segment in any jawed vertebrate species analyzed.

In order to infer whether the vertebrate ancestor already possessed CTCF binding sites between Hox1 and -4 (see Results), we attempted to infer *in silico* the CTCF binding landscape in the Hox cluster of amphioxus, as well as the elephant shark, using the FIMO program, because ChIP-seq data on CTCF was not available for these species. The prediction referring to the motifs of the species analyzed with ChIP-seq data in this study, however, detected only a few CTCF binding sites inside the amphioxus and elephant shark Hox clusters. In fact, this negative result was not validated, as this method alone did not accurately predict CTCF binding sites in the human Hox clusters, either. The only species with CTCF ChIP-seq data that potentially serves as an outgroup is the fruitfly. In its *Antennapedia* complex (ANT-C), we identified outstanding ChIP-seq peaks between *pb* and *Dfd* genes, orthologous to vertebrate Hox2 and Hox4 genes, respectively (Supplementary Fig. S8).

## Discussion

**Figure 5:**
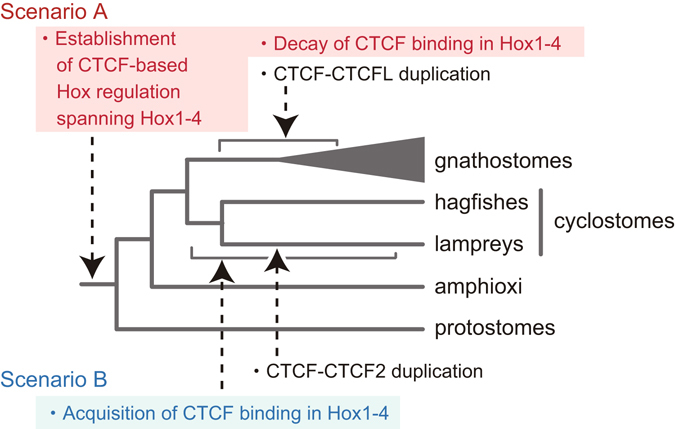
Possible scenarios of the establishment of CTCF binding patterns in vertebrate Hox clusters. Alternative evolutionary scenarios of CTCF gene duplication and CTCF binding patterns in Hox clusters are depicted. CTCF-based Hox regulation spanning Hox1-4 genes was established either at the ancestor of bilaterians (Scenario A) or in the cyclostome lineage (Scenario B).

The present study encompassed the whole phylogeny of vertebrate CTCF and its relatives, now including the jawless vertebrate homologs. Out of the two lamprey CTCF genes identified in our gene model GRAS-LJ, *LjCTCF* is thought to be the canonical *CTCF* ortholog because it is expressed generally at higher levels and possesses the same number of Zn finger domains in its protein product as its gnathostome counterparts (Fig. 1). *LjCTCF2* was revealed to be relatively lowly expressed and more rapidly-evolving, retaining fewer Zn finger domains (Fig. 1). Importantly, our in-depth phylogenetic analysis ruled out the possibility that lamprey *CTCF2* is orthologous to *CTCFL*, and rather suggested the origin of *CTCF2* in the lamprey lineage (Fig. 5). We also propose that *CTCFL* arose through gene duplication before the split of the chondrichthyan lineage (Fig. 1d and Supplementary Fig. S3). The hypothesis by Hore *et al*., postulating the origin of *CTCFL* early in amniote evolution^22^, was not statistically rejected (*p* ≥ 0.069; Table 2) and is preferred by maximum parsimony principle as it assumes fewer gene losses. However, Hore *et al*. inferred the phylogenetic tree employing a simple evolutionary model (*p*-distance based on nucleotide substitutions) and tree inference method (neighbor-joining method). We inferred phylogenetic trees using a similar sequence set as Hore *et al*. but employing more modern phylogenetic approaches, which did not suggest a recent duplication timing (Supplementary Fig. S3e,f,k and l). Tree inference using coding nucleotide sequences may cause phylogenetic mispositioning because the third codon positions lose phylogenetic signatures if substitutions are saturated. Indeed, our analysis using peptide sequences or nucleotides in the first and second codon positions favored a more ancient origin of *CTCFL* (Supplementary Fig. S3a–d and g–j).

Our ChIP-seq experiments for lamprey CTCF were enabled by validation of antibody specificity across species and our optimized ChIP protocol. Our results on the lamprey revealed high similarities in the number and distribution of ChIP-seq peaks between different tissues or between different life stages (e.g., between liver and embryos; Fig. 3b and c), as previously shown for human^24, 25^. This observation validates the cross-species comparison of CTCF ChIP-seq peak distribution, even between different tissues and between embryonic stages. Our genome-wide comparison revealed a high similarity in numbers of CTCF binding sites, in spite of their variable genome sizes (1.2 Gbp for chicken, 1.6 Gbp for the Arctic lamprey, and 3.5 Gbp for human)^15^, as well as in the core and upstream binding motif sequences between Arctic lamprey, chicken, and mammals (Fig. 3b,c and d). We conclude that the establishment of CTCF binding property, known for mammals, occurred in the last common ancestor of extant vertebrates at the latest.

Distribution of ChIP-seq peaks in lamprey was shown to be associated with that of repetitive sequences, particularly retrotransposons and DNA transposons (Table 3; Supplementary Fig. S9). It was previously reported that CTCF binding sites are associated with retrotransposons in mammalian genomes^3, 8^. These suggest a similar process of CTCF binding site propagation within a genome, mediated by transposons, occurring independently in these different lineages.

A recent analysis suggested that some epigenomic features of mammalian Hox clusters, such as their global regulation involving distal enhancers, had not been established at the chordate ancestor^28^. In mice, the outward CTCF binding motif located between HoxA5 and HoxA6 functions as an anchor point for long range interaction^12^. Studies on cyclostomes should thus help pinpoint the timing of its establishment. Whole-genome sequencing on lampreys previously showed the elongation of Hox clusters^14, 15^, while their precise structures have not yet been determined. The present study provided a comparative landscape of repetitive sequences in Hox clusters (Supplementary Table S6), which revealed the contrast that lamprey Hox clusters are massively intruded by repetitive elements, whereas the Hox clusters of the gnathostomes analyzed are almost devoid of them. The increase in the number of CTCF binding sites inside the lamprey Hox clusters might be explained by transposon-based CTCF binding site propagation and maintenance (Fig. 4). Indeed, 72.7% (40 out of 55) of CTCF ChIP-seq peaks inside the Hox α-ε clusters were associated with repeat elements, which was similar to the proportion of their genome-wide association (72.3%; 19,981 out of 27,633 CTCF peaks) (Supplementary Fig. S9).

While the exact structures of lamprey Hox clusters remain to be determined, our CTCF ChIP-seq data has enabled the first vertebrate-wide comparison of CTCF binding patterns in Hox clusters. The shared positioning of the binding sites between different clusters might suggest that they originated before the split of the clusters. It showed that there is a common outward orientation of CTCF binding sites closest to the ends of Hox clusters, in both lamprey and gnathostomes (Fig. 4). Most striking was the contrast in the region between Hox1 and Hox4 intruded by CTCF binding sites in the lamprey, whereas no or few binding sites were identified in this segment in all the gnathostomes analyzed (Fig. 4 and Supplementary Fig. S6). The identification of CTCF motifs in the corresponding locations in the fruitfly Hox cluster (Supplementary Fig. S8) suggests an early origin of CTCF binding in the Hox1-4 region at the bilaterian ancestor, and that they subsequently decayed in the jawed vertebrate lineage (Scenario A in Fig. 5). On the other hand, Scenario B in Fig. 5 is supported by a notion that repeat-associated propagation of CTCF binding motifs might have occurred relatively recently, in light of the similar event in the mammalian lineage that occurred within these 100 million years at most.

Hox genes regulate the morphogenesis of various tissues in vertebrate embryos, and the origins of its molecular mechanisms have been intensively analyzed in lampreys, focusing on the hindbrain^29, 30^, pharyngeal arches^31^, and axial elements in the trunk^32^. It is of great interest to characterize possible influence of CTCF binding in the genomic region spanning Hox1 to Hox4 on morphological evolution marking the distinction between cyclostomes and the rest of the vertebrates, such as the acquisition of the articulating jaw. To address this point, it is crucial to characterize CTCF binding in chondrichthyan Hox clusters, which is currently ongoing.

## Materials and Methods

### Animals and Cells

Tissues of brain, eye, heart, intestine, liver, skeletal muscle, and pooled oocytes of an adult female, and the testis of an adult male Arctic lamprey, *L. camtschaticum*, from the Shiribetsu river, Hokkaido, Japan, were dissected, snap frozen in liquid nitrogen, and kept at −80 °C until use. *L. camtschaticum* embryos obtained by artificial fertilization were sampled as pools at stages 25 and 27, according to the embryonic staging of *L. reissneri* ^33^ and stored as above. Fertilized chicken eggs were obtained from a local farm. Chicken embryos used were at stage 25 according to the embryonic staging by Hamburger and Hamilton^34^, snap frozen, and kept at −80 °C until use. Human lymphoblastoid cell line GM12878, was purchased from the Coriell Cell Repositories and cultured in RPMI-1640 media (Gibco) supplemented with 15% FBS, 2 mM L-glutamine, and 1x antibiotic-antimycotic solution (Gibco), at 37 °C, 5% CO_2_. Animal care and experimental procedures were conducted in accordance with guidelines approved by the Institutional Animal Care and Use Committee (IACUC), RIKEN Kobe Branch.

### RNA Extraction and RNA-seq Library Preparation

Tissues and embryos of *L. camtschaticum* were powderized while frozen, using SK-200 mill (Tokken) with stainless tubes and bullets. Tissue powders were quickly lysed in Trizol reagent (Thermo Fisher Scientific) to extract total RNAs. Libraries for RNA-seq were prepared using 1 μg total RNA following the standard protocol of TruSeq Stranded mRNA Sample Prep Kit (Illumina).

### Whole-Mount *In Situ* Hybridization

Whole-mount *in situ* hybridization was performed following previous literature^35^. Riboprobes were designed for 5′ and 3′ regions of the *LjCTCF* and *LjCTCF2* transcript sequences (Supplementary Fig. S10) and prepared using cDNA of embryos at stage 27 with oligonucleotide primers in Supplementary Table S7.

### Molecular Phylogenetic Analysis

Deduced amino acid sequences of *CTCF*, *CTCFL*, and *CTCF2* genes were aligned with the program MAFFT v7.221^36^ employing the L-INS-i method. From the entire multiple alignment, the stretch of Zn finger domains was extracted, and ambiguously aligned sites were removed with trimAl v1.4^37^ with the ‘-automated1’ option followed by removal of gapped sites. Molecular phylogenetic trees with the maximum-likelihood (ML) framework were inferred using the program RAxML v8.2.4^38^ assuming the PROTCATWAG model for amino acid sequences and the CATGTR model with assignment of distinct models to the codon positions for nucleotides. Credibility of the nodes in ML trees was evaluated with 1,000 bootstrap resamplings employing the rapid bootstrapping implemented in RAxML. Phylogenetic trees with the Bayesian framework were inferred with PhyloBayes v4.1b^39^ assuming the CAT-WAG-Γ model for amino acid sequences and the CAT-GTR-Γ model for nucleotide sequences. Tree-topology test was performed with a combination of CONSEL^40^ and RAxML assuming the PROTGAMMAWAG model. Accession numbers and sequences used for the analysis are listed in Supplementary Tables S8 and S9.

### Chromatin Immunoprecipitation

Frozen liver tissue from an adult female lamprey, lamprey embryos at stage 27, and chicken embryos at stage 25, were powderized using a SK-200 mill and fixed in 1% formaldehyde/PBS (-) solution for 15 minutes at room temperature. Human GM12878 cells were fixed in 1% formaldehyde/PBS (-) solution for 5 minutes at room temperature. Chromatin lysates were prepared in lysis buffer (50 mM Tris-HCl pH8.0, 1% SDS, 10 mM EDTA, 1% proteinase inhibitor), by sonication with Covaris S220 or E220 (COVARIS).

ChIP was performed in ChIP dilution buffer (16.7 mM Tris-HCl (pH 8.0), 0.01% SDS, 1.1% (w/v) TritonX-100, 1.2 mM EDTA, 167 mM NaCl, 1% proteinase inhibitor) containing 4 mg/ml BSA (for lamprey and chicken embryonic samples) or 0.5 mg/ml BSA (for lamprey liver and human GM12878 cells) using lysates equivalent to 1 × 10^7^ cells and protein A beads (Novex) coupled with 5 μl of anti-CTCF antibody (CST #3418 S). After 4 hours of IP reaction at 4 °C, beads were washed 4 times in a low salt buffer (20 mM Tris-HCl (pH 8.0), 0.1% SDS, 1% (w/v) TritonX-100, 2 mM EDTA, 150 mM NaCl), and two more times with a high salt buffer (20 mM Tris-HCl (pH 8.0), 0.1% SDS, 1% (w/v) TritonX-100, 2 mM EDTA, 500 mM NaCl). Chromatin complexes were eluted from the beads by agitation in elution buffer (10 mM Tris-HCl (pH 8.0), 300 mM NaCl, 5 mM EDTA, 1% SDS) and incubated overnight at 65 °C for reverse-crosslinking. Eluates were treated with RNase A and Proteinase K, and ChIP DNA was ethanol precipitated. Libraries were prepared using 20 ng of input DNA and 1 ng of ChIP DNA with KAPA LTP Library Preparation Kit (KAPA Biosystems) and custom synthesized TruSeq adaptors. The procedure of ChIP and library preparation is detailed in SI Materials and Methods.

### Sequencing

All RNA-seq libraries and all but two ChIP-seq libraries were subjected to on-board cluster generation using HiSeq SR Rapid Cluster Kit v2 (Illumina) and sequenced on Rapid Run Mode of Illumina HiSeq 1500 (Illumina) to obtain single end 80 nt reads. Two ChIP-seq libraries (GM12878_input_rep1 and GM12878_ChIP_rep1) were subjected to cluster generation in cBot using TruSeq PE Cluster Kit v3 cBot-HS (Illumina) and sequenced in the high-output mode of HiSeq 1500 to obtain paired-end 101 nt reads. Only the forward reads from the paired-end data were used for analysis after trimming them to 80 nt, to adjust to those of the other samples. All the RNA-seq and ChIP-seq reads were processed by Trim Galore! v0.3.7 (http://www.bioinformatics.babraham.ac.uk/projects/trim_galore/) at default parameters to trim reads at low quality sequences and Illumina TruSeq adaptor sequences. RNA-seq and ChIP-seq data are available at DDBJ Sequence Read Archive (DRA) under the accession DRA005605.

### ChIP-seq Data Analysis

Arctic lamprey, chicken, and human ChIP-seq reads were mapped to the LetJap1.0, galGal5, and hg19 genome assemblies using Bowtie v0.12.8^41^, respectively. For lamprey and chicken, uniquely mapped reads were obtained using ‘-m 1 -v 2 -a --best --strata’ options. Subsequently, unmapped reads were mapped using ‘-m 5 -n 2 -a --best --strata’ options, allowing each read to align with up to 5 locations, and combined with the uniquely mapped reads. For human, only uniquely mapped reads were obtained using ‘-m 1 -n 2 -a --best --strata’ options. Peak calling was performed using MACS2 v2.0.10 with default parameters, including a *q*-value cutoff of 0.01. Each of ChIP replicate 1, ChIP replicate 2, and a merger of these two replicates (“merged replicates”) was used as a treatment sample, while input replicate 1 was used as a control sample in all the three peak calling runs. Intersect peaks in replicate 1, replicate 2, and “merged replicates” identified by Bedtools v2.17.0^42^, were annotated as “consensus peaks”. A subset of “consensus peaks” with their fold enrichment for “merged replicates” of ≥10 (for lamprey and chicken) and ≥20 (for human) was assigned as “significant peaks”. These enrichment cutoffs were determined referring to the averages of fold enrichment among “consensus peaks” of 16.2, 15.2, 13.3, and 29.1 for lamprey embryos, lamprey liver, chicken embryos, and human GM12878 cells, respectively (Supplementary Table S5).

Motif identification was performed using MEME v4.10.0. The top 2,000 peaks, ranked by fold enrichment, were used to identify enriched sequence motifs within ± 100 bp from the peak summit. Background 1st-order Markov model was made from the Top 2,000 peaks (summit ± 100 bp) with ‘-pseudo 0.01’ option. After identifying the 16 bp-long core CTCF motif by MEME, FIMO v4.10.1^43^ was used with ‘--thresh 1e-4 and --motif-pseudo 0.01’ options to identify motif locations in all the peak regions (summit ± 100 bp). The core CTCF motif sequences identified by FIMO were extended for 20 bp to the 5′ direction to search for the previously described ‘upstream motif’ (also called ‘M2 motif’)^3, 4^. The analysis for ‘upstream motif’ was performed similarly to the core motif analysis, motif identified by MEME and motif located by FIMO with ‘--thresh 1e-3 and --motif-pseudo 0.01’ options.

### Association of CTCF Binding to Repetitive Elements

RepeatModeler^44^ was ran on the genome assembly LetJap1.0 with default parameters, which resulted in 847 repeat elements including SINE (49 entries), LINE (202 entries), LTR (82 entries), and DNA transposon (145 entries). Positions of repeat elements were obtained by running RepeatMasker v4.0.5^45^ against the LetJap1.0 genome using the custom repeat library obtained above.

Overlaps of CTCF binding sites with the repeat elements were analyzed with Bedtools targeting 41,421 consensus peaks of 100 bp (summit ± 50 bp) from the stage 27 embryo CTCF ChIP-seq data with fold enrichment of no less than 5. As a control to assess the significance of association with repeats, we arbitrarily selected from the LetJap1.0 genome assembly 50,000 non-overlapping genomic regions of 100 bp with no undetermined base (‘N’) inside. CTCF binding sites overlapping each of the 847 repeat elements were counted and tested for statistical significance by Fisher’s exact test. *p*-values were subjected to multiple correction by the Q-value package v1.34.0^46^ implemented in R.

### Comparison of CTCF Binding in Hox Clusters

Only “significant peaks” in CTCF ChIP-seq results were used in cross-species comparisons. The location and orientation of CTCF motifs within peak regions (summit ± 100 bp) were identified by FIMO with ‘--thresh 1e-3 and --motif-pseudo 0.01’ options. When multiple motifs were identified within a peak, only the motif with the lowest *p*-value was adopted for downstream analyses. The CTCF motif and the background file used here were those identified from the top 2,000 peaks for each species.

### Data Availability

Nucleotide sequences of the *L. camtschaticum LjCTCF* and *LjCTCF2* cDNAs were deposited in the NCBI GenBank database under the accession numbers KX830966 and KX830967, respectively. RNA-seq and ChIP-seq data were deposited in the DDBJ DRA under the accession DRA005605.

## Electronic supplementary material

Supplementary Info
