# Supplementary material for: CTCF binding landscape in jawless fish with reference to Hox cluster evolution
Source: Sci Rep. 2017 Jul 10;7:4957. doi: 10.1038/s41598-017-04506-x (PMC5504073; doi:10.1038/s41598-017-04506-x)
Supplement: Supplementary file 1 — Supplementary Info [file 41598_2017_4506_MOESM1_ESM.pdf]

Supplementary Information

CTCF binding landscape in jawless fish with reference to Hox cluster evolution

Mitsutaka Kadota[1], Yuichiro Hara[1], Kaori Tanaka[1], Wataru Takagi[2], Chiharu Tanegashima[1], Osamu Nishimura[1] & Shigehiro Kuraku[1]

[1]Phyloinformatics Unit, RIKEN Center for Life Science Technologies, 2-2-3 Minatojima-minami, Chuo-ku, Kobe, 650-0047, Japan.

[2]Evolutionary Morphology Laboratory, RIKEN, 2-2-3 Minatojima-minami, Chuo-ku, Kobe, 650-0047, Japan.

Yuichiro Hara, Kaori Tanaka and Wataru Takagi contributed equally to this work. Correspondence and requests for materials should be addressed to S.K. (email: shigehiro.kuraku@riken.jp)

Materials and Methods

cDNA Cloning and Sanger Sequencing. For *LjCTCF*, cDNA was synthesized from total RNA extracted from pooled stage 27 embryos, using SuperScript III Reverse Transcriptase (Thermo Fisher Scientific) and a gene specific primer. RT-PCR was performed to amplify cDNA containing the full-length ORF of approximately 3.7 Kbp using KAPA HiFi HotStart ReadyMix (KAPA Biosystems) and 1 M Betaine (Sigma) in the reaction. The PCR product was ligated into the pCR4Blunt-TOPO vector (Thermo Fisher Scientific) and sequenced on a 3730xl DNA Analyzer (Thermo Fisher Scientific).

For *LjCTCF2*, 3′RACE was performed using total RNA extracted from pooled stage 25 embryos, with 3′ RACE kit (Thermo Fisher Scientific) and gene specific primers. Total RNA extracted from pooled stage 25 embryos was reverse transcribed into cDNA, with ThermoScript Reverse Transcriptase (Thermo Fisher Scientific) and a primer specific to *LjCTCF2*. RT-PCR was performed to amplify cDNA containing the putative full-length ORF of approximately 1.3 Kbp using Phusion High-Fidelity DNA Polymerase (NEB) and the supplied GC buffer. PCR products were A-tailed using Taq HS Perfect Mix (Takara Bio), ligated into the pCRII-TOPO vector (Thermo Fisher Scientific) and sequenced as above. Oligonucleotide primers used here are included in Supplementary Table S7.

Western Blotting, Immunoprecipitation and Mass Spectrometry. Frozen tissues and embryos were powderized using SK-200 mill (Tokken) and lysed in RIPA buffer containing 1 % protease inhibitor solution (Sigma, P8340) and 1 mM DTT.

For western blotting, 20-30 μg of total proteins were electrophoresed, transferred to a nitrocellulose membrane using iBlot (Thermo Fisher Scientific) for Fig. 2a left, and a PVDF membrane by wet electroblotting method for Fig. 2a right, and blocked with 5 % skim milk. Antigen was detected by incubating the membranes at 4 °C in TBST buffer using either anti-CTCF antibody (CST #3418S, 1:2000 dilution), anti-β-actin antibody (CST #5125, 1:2000 dilution), or anti-histone H3 antibody (Wako #304-34781, 1:2000 dilution). HRP-conjugated anti-rabbit IgG secondary antibody or HRP-conjugated anti-mouse IgG secondary antibody (1:2000 dilution) was used to detect the primary antibodies by the ECL system (GE Healthcare Life Science).

Immunoprecipitation was carried out using 1-3 mg of protein lysate and 10 μl of anti-CTCF antibody in RIPA buffer at 4 °C for 4 hours under gentle rotation. Following the addition of Protein A beads (Thermo Fisher Scientific), the reaction mixture was incubated for another 1 hour with rotation at 4 °C. Beads were washed with RIPA buffer three times, and proteins were eluted and denatured by boiling the beads in SDS sample buffer for 5 minutes. Following electrophoresis, proteins were stained with silver staining kit (Wako). Gel bands were excised and analyzed by mass spectrometry (LC-MS/MS) as described previously[1].

Gene Prediction and Curation. Previous genome analysis on *L. camtschaticum* did not provide gene annotation to be readily used in RNA-seq and ChIP-seq data analysis[2]. For this reason, we performed gene prediction and its evidence-based improvement as follows (see Supplementary Fig. S1, for a flow chart of this entire procedure). First, the program BUSCO v1[3] was ran on the *L. camtschaticum* genome assembly LetJap1.0, referring to the metazoan gene set (mBUSCO), which resulted in 535 mBUSCO component genes identified on the genomic scaffold sequences that were longer than 100 Kbp. To increase sequence-level heterogeneity among them, we ran blastclust[4] with options '-L 0.1 -S 20'. In order for individual genes to represent distinct genomic regions with their flanking non-coding sequences, we discarded one of the neighboring gene pairs that are located with an intergenic sequence shorter than 3 Kbp. 450 genes that remained after these selections were used in a training of the gene prediction program AUGUSTUS v3.1[5], according to the developer's instruction (http://bioinf.uni-greifswald.de/augustus/binaries/tutorial/training.html). Second, in order to improve the accuracy of identification of exon-boundaries later by AUGUSTUS, we aligned RNA-seq reads to the genome assembly (LetJap1.0) using TopHat2 v2.0.11[6] and Bowtie2 v2.2.2[7] with the default setting without providing any gene model, and constructed 'intron hints' according to the developer's instruction (http://bioinf.uni-greifswald.de/bioinf/wiki/pmwiki.php?n=IncorporatingRNAseq.Tophat) (see Supplementary Table S1 for mapping rates). In addition, in order to generate 'CDSpart hints' based on homologs of other species, a set of vertebrate protein sequences were used as queries in TBLASTN with a threshold of 1e-10, followed by exact identification of protein-coding regions with exon-intron boundaries taken into account using Exonerate v2.2.0[8]. The vertebrate protein sequence set consisted of RefSeq 'known' proteins of human (39,582 sequences), chicken (6,189 sequences), and amniote vertebrates (44,675 sequences), as well as predicted sea lamprey genes (24, 271 sequences[9]). Third, the LetJap1.0 genome assembly was subjected to identification of conventional and *de novo* repetitive sequences with RepeatModeler v1.0.4[10] with default parameters, followed by masking of the identified repetitive sequences with RepeatMasker v4.0.5[11] with the options '-nolow -xsmall'. Fourth, the program AUGUSTUS was run with the trained parameter files on the genome assembly in which the identified repeats are soft-masked, incorporating intron hints and CDSpart hints with the options '--singlestrand=false --alternatives-from-evidence=false --allow_hinted_splicesites=atac --softmasking=1'.

These predicted genes were then further improved by making use of transcriptional evidence from RNA-seq, as well as sequence similarity to the aforementioned vertebrate protein sequence set. The RNA-seq reads were used to build a transcriptome assembly via mapping-first and assembly-first approaches. In the mapping-first approach, RNA-seq reads were first aligned to the Arctic lamprey genome assembly with Tophat2 v2.0.11. This alignment data was used in combination with the AUGUSTUS predicted gene model to generate a transcriptome assembly by Cufflinks v2.2.1[12]. In the assembly-first approach, the same RNA-seq reads were utilized by Trinity r20140413p1[13] to produce *de novo* transcriptome assemblies, which were aligned to the Arctic lamprey genome with

PASA v2.0.2[14]. The coding regions of the assembled transcripts from both approaches were deduced using Transdecoder v2.0.1[15], which employed homology matches to the UniProtKB/Swiss-Prot release 2014_07 peptides. Once this was completed, the transcripts with putative ORFs were collected, and their deduced amino acid sequences were compared against their best-hit match to the vertebrate proteins. From this comparison, a protein-coding transcript evidence set, consisting of the assembled transcripts which had at least 80% of their length matching to at least 80% of a RefSeq protein, was obtained.

The transcript evidence set was aligned back against the AUGUSTUS predicted genes. From this analysis, certain AUGUSTUS genes, depending on how they lined up against the transcript evidence, were placed into one of four different categories, (i) split genes, (ii) fusion genes, (iii) truncated genes, and (iv) unannotated genes (see Supplementary Fig. S1b). (i) A split gene had the putative ORF of the transcript evidence overlapping with more than two AUGUSTUS genes; if these AUGUSTUS genes matched to different parts of the same vertebrate protein, they were replaced by the exons composing the transcript evidence. (ii) A fusion gene occurred when multiple vertebrate protein-genome alignments, which were generated with Exonerate as described above, overlapped with different parts of a single AUGUSTUS gene; if at least 60% of the individual protein-genome alignments uniquely overlapped with at least 60% of the transcript evidence in different AUGUSTUS gene positions, the AUGUSTUS gene was replaced by the exons of the overlapping transcript evidence. (iii) A truncated gene was specified as an AUGUSTUS gene that was entirely overlapped by a coding sequence in the transcript evidence; the AUGUSTUS gene was replaced with the exons composing the transcript evidence if its match to the vertebrate protein was longer than the AUGUSTUS gene's match to the vertebrate protein. (iv) An unannotated gene was a case where at least 60% of a vertebrate protein aligned to a region of the genome in which no genes were predicted by AUGUSTUS; if a coding region in the transcript evidence overlapped with the vertebrate protein, the exons composing the transcript evidence was added to the gene set. In each of these cases, similarity searches of the amino acid sequences of both the AUGUSTUS genes and the assembled transcripts against the vertebrate proteins were performed using FASTA v36.3.8b[16], with the numbers of replaced genes in each category shown in Supplementary Table S2. As a result, a total of 34,435 genes were identified. The completeness of the gene set assessed by BUSCO increased by 1 after the procedure for correcting mispredictions outlined above (Supplementary Table S2). The produced gene model is available at our laboratory web site (http://www2.clst.riken.jp/phylo/GRAS-LJ.gff3.gz).

Gene Expression Quantification using RNA-seq Data. Gene expression quantification was performed by mapping RNA-seq reads to nucleotide coding sequences of the genes predicted as above, except that incompletely predicted *LjCTCF* and *LjCTCF2* sequences were replaced with their exonic nucleotide sequences containing their full ORFs that we confirmed with cDNA cloning. Using these sequences as reference, read mapping was performed with Bowtie2 v2.2.2 with '--very-

sensitive-local' option followed by FPKM calculation by eXpress v1.5.1[17]. See Supplementary Table S1 for mapping rates.

ChIP-seq Data Analysis. ChIP-seq data for mouse E14.5 embryonic brain (SRR392354 and SRR505014), E14.5 MEF (SRR207080 and SRR207071), and ES cells (SRR207089 and SRR207081) were obtained from NCBI SRA. ChIP-seq data for adult dog liver (ERR022285, ERR022304) and adult opossum liver (ERR022303, ERR022307, ERR022306, and ERR022301) were obtained from EBI ENA. ChIP-seq data for fly *Drosophila melanogaster* (SRR066831-SRR066836), was obtained from NCBI SRA. ChIP-seq reads were processed by Trim Galore! v0.3.7 (http://www.bioinformatics.babraham.ac.uk/projects/trim_galore/) with default parameters except for the opossum data. ChIP-seq reads for mouse, dog, opossum, and fly were mapped to mm10, CanFam3.1, MonDom5, and Dmel r6.11 genome assemblies using Bowtie v0.12.8[18], respectively. For mouse and fly data, only uniquely mapped reads were obtained using the options '-m 1 -n 2 -a --best --strata'. For dog and opossum data, uniquely mapped reads were first obtained using the options '-m 1 -v 2 -a --best --strata'. Subsequently, unmapped reads were mapped using the options '-m 5 -n 2 -a --best --strata', allowing each read to align with up to five locations, and combined with the uniquely mapped reads. Peak calling was performed using MACS2 v2.0.10[19] with its default parameters. Identification of the CTCF 'core' and 'upstream' motifs was performed for mouse, dog, and opossum as described in Materials and Methods, using sequences from the top 2000 peak regions. Only the 'core motif' was identified in the top 500 peak regions for the fly. The location and orientation of CTCF motifs within peak regions inside Hox clusters were identified by FIMO v4.10.1[20] as described in Materials and Methods.

Protein Identification by Chromatography-coupled Tandem Mass Spectrometry (LC-MS/MS). Proteins from gel slices were subjected to reduction/alkylation, followed by digestion with 10 μg/ml trypsin at 37 °C for 16 hours. Peptides were extracted with 5 % formic acid and 50 % acetonitrile, dried, and dissolved in 2 % acetonitrile and 0.1 % formic acid. Peptides were fractionated by reverse-phase chromatography (ADVANCE UHPLC) and applied to ion trap mass spectrometer (LTQ Orbitrap Velos Pro; ThermoFisher) with advanced captive spray source. The Mascot software (Matrix Science) was used to identify the match of molecular masses to non-redundant protein from the NCBI database (ver. 20151116), while for *L. camtschaticum*, peptides were searched against the gene set predicted by AUGUSTUS from the *L. camtschaticum* genome (LetJap1.0) containing the CTCF protein sequences determined in this study.

Chromatin Immunoprecipitation Protocol. Chromatin immunoprecipitation was performed basically as described[21] but with modifications to adapt to embryos and tissues of the Arctic lamprey

L. camtschaticum and chicken.

Step-1: Sample Preparation. Lamprey embryos were collected and pooled at stage 27. The lamprey liver tissue was obtained from an adult female after induced insemination. Chicken embryos were sampled at stage 25. Tissue and embryo samples were snap frozen in liquid nitrogen after dissection and stored at −80 °C. The GM12878 cell line was cultured in RPMI-1640 medium (Gibco, 21870-076) supplemented with 15 % heat inactivated FBS (Gibco, 10082-139), 1× L-glutamine (Gibco, 25030081) and 1× Antibiotic-antimycotic solution (Gibco, 15240-096), at 37 °C under 5 % CO_2.

Step-2: Cell Fixation. The SK-200 frost mill with metal tubes and bullets was used to manually powderize 100 mg of lamprey liver tissue, 100 mg of pooled lamprey embryos, and a whole chicken embryo. The pulverized samples were transferred to frozen 2.0 ml tubes and dissolved in 1.5 ml PBS (−) supplemented with 1 % formaldehyde (Pierce, 28906). Fixation was performed for 15 minutes at room temperature with gentle agitation. Approximately 1× 10^8 GM12878 cells were fixed in 10 ml of 1 % formaldehyde solution for 5 minutes at room temperature with gentle agitation. Glycine was added to a final concentration of 0.125 M in order to quench the crosslinking reaction. Samples were centrifuged, and washed twice with cold PBS (−).

Step-3: Lysate Preparation. Fixed samples were resuspended in either 1 ml (for the liver tissue and embryos) or 10 ml (for GM12878 cells) of chilled LB1 buffer [50 mM Hepes-KOH, pH 7.5, 140 mM NaCl, 1 mM EDTA, 10 % (w/v) Glycerol, 0.5 % (w/v) NP-40, 0.25 % (w/v) TritonX-100, 0.1% protease inhibitor (SIGMA, P8340)], and incubated at 4 °C for 10 minutes with gentle agitation. Tissue and embryo samples were subjected to 10~20 strokes of dounce homogenization on ice with the tissue grinder set pestle B (Sigma, D8938). After centrifugation, cells were washed once in either 1 ml (for the liver tissue and embryos) or 10 ml (for GM12878 cells) of chilled LB2 buffer [1 mM Tris-HCl pH 8.0, 200 mM NaCl, 1 mM EDTA, 0.5 mM EGTA, 0.1% protease inhibitor (SIGMA, P8340)], followed by 3 additional washes in 1 ml of chilled LB3 buffer [50 mM Tris-HCl pH8.0, 1 % SDS, 10 mM EDTA, 1% protease inhibitor (SIGMA, P8340)]. Tissue and embryo samples were resuspended in 130, 260, or 390 µl of LB3 buffer, depending on the amount of cells used. GM12878 cells were resuspended in 1 ml of LB3 buffer. Chromatin lysates were prepared by sonication in Covaris S220 or E220 (COVARIS). Tissue and embryo chromatin lysates were prepared at PIP 140, 5 % duty cycle, 200 cycles per burst, 7 °C bath temperature, and 16-minute duration in 130 µl/microTUBE. Chromatin lysate of GM12878 cells was prepared at PIP 140, 10 % duty cycle, 200 cycles per burst, 7 °C bath temperature, and 20-minute duration in 1 ml/milliTUBE. Debris was removed by centrifugation at 20,000 ×g for 5 minutes at 4 °C. Lysates in the supernatant were snap frozen in liquid nitrogen and kept at −80 °C for later use.

Step-4: Assessment of Chromatin DNA Fragmentation. To assess the degree of chromatin DNA fragmentation, 10 µl of lysates were diluted in 200 µl of Elution buffer (10 mM Tris-HCl pH8.0, 300 mM NaCl, 5 mM EDTA, 1 % SDS) and incubated at 65 °C for more than 6 hours to reverse the crosslinking. Samples were further treated with 10 µg of RNase A for 20 minutes at

37 °C, and with 100 µg of Proteinase K for 40 minutes at 55 °C. Samples were transferred to 1.5 ml DNA LoBind tubes (Eppendorf, 0030108051), mixed and vortexed with 200 µl of Phenol:Chloroform:Isoamyl Alcohol solution (25:24:1, v/v) and centrifuged at 20,000 ×g for 5 minutes at room temperature. Subsequently, about 180 µl of the aqueous phase was transferred to a new DNA LoBind tube, after which 200 µl of TE buffer (containing 300 mM NaCl) was added to the remaining organic phase. The aqueous phase extraction was repeated, and about 200 µl of the 2nd aqueous phase was combined with the first aqueous phase. Ethanol precipitation was carried out by adding 20 µg of glycogen and 900 µl of ice cold 100 % ethanol. DNA was precipitated by centrifugation at 20,000 ×g for 30 minutes at 4 °C. The DNA pellet was washed once in 1 ml of 75 % ethanol, and centrifuged again at 20,000 ×g for 5 minutes at 4 °C to remove all traces of ethanol. The DNA pellet was air dried for 2 minutes at room temperature with the tube lid open, and dissolved in 50 µl of TE solution. DNA Concentration was measured using Qubit dsDNA HS Assay kit (Thermo Fisher Scientific, Q32851), and size distribution was measured using Agilent High Sensitivity DNA kit (Agilent Technologies, 5067-4626). Chromatin fragmentation was considered optimal if 1) more than 80 % of DNA fragments were within ranges of 100-500 bp, and 2) the average size of DNA fragments were between 200-300 bp. Concentrations of lysates in terms of cell numbers were estimated from the total amount of input DNA obtained from 10 µl of each of the chromatin lysates, using factors of 6 pg DNA, 2 pg DNA, and 3 pg DNA per cell for human, chicken and lamprey, respectively.

Step-5: IP Reaction. Chromatin immunoprecipitation (ChIP) was performed using lysates equivalent to 1×10^7 cells. Chromatin lysates were diluted 10 folds in ChIP dilution buffer (16.7 mM Tris-HCl (pH8.0), 0.01 % SDS, 1.1 % (w/v) TritonX-100, 1.2 mM EDTA, 167 mM NaCl, 0.1% protease inhibitor) containing 0.5 mg/ml BSA (for lamprey liver and GM12878 cells) or 4 mg/ml BSA (for lamprey and chicken embryos). Protein A beads (Novex, 10002D) were coupled with 5 µl of anti-CTCF antibody (CST, #3418 S) in ChIP dilution buffer for 1 hour at 4 °C. Antibody-coupled beads were washed once with ChIP dilution buffer containing 0.5 mg/ml BSA, added to the diluted lysate, and incubated for 4 hours at 4 °C under gentle agitation. After the IP reaction, beads were washed 4 times in low salt buffer (20mM Tris-HCl (pH 8.0), 0.1 % SDS, 1% (w/v) TritonX-100, 2mM EDTA, 150 mM NaCl), and twice in high salt buffer (20 mM Tris-HCl (pH8.0), 0.1 % SDS, 1 % (w/v) TritonX-100, 2mM EDTA, 500 mM NaCl). The immune complexes were eluted from the beads by agitation in elution buffer (10 mM Tris-HCl (pH8.0), 300 mM NaCl, 5 mM EDTA, 1 % SDS) at room temperature for 15 minutes, and supernatants were transferred to a new tube and incubated overnight at 65 °C for reverse-crosslinking. Eluates were further treated with RNase A and Protease K, and they were precipitated in ethanol to obtain ChIP DNA, as described for the input DNA.

Step-6: ChIP DNA QC. Prior to library preparation, enrichment of the CTCF bound regions in the ChIP DNA was analyzed by quantitative qPCR using primers designed for human *H19*, *HoxA6* and *Gapdh* loci, for chicken *HoxB8*, *HoxD8*, and *Actb* loci, and for lamprey *Hox-α5*, *Hox-α6* and *Actb*

loci. CTCF binding sites in human were identified by analyzing ChIP-seq datasets from a public database, while for chicken and lamprey, CTCF binding sites were predicted using the database JASPAR 2014 (http://jaspar.genereg.net/). Enrichment folds were calculated as the ratio between "quantitated concentrations by qPCR" and "actual DNA concentration". Samples that displayed enrichment greater than 10 fold were used for library preparation.

Step-7: Library Preparation and Library QC. Libraries were prepared from 20-30 ng input DNA or 1-5 ng ChIP DNA using KAPA LTP Library Preparation Kit (KAPA Biosystems, KK8232) with TruSeq index adaptors. We modified the manufacturer's protocol and carried out reaction at 1/5 scale. The amount of PEG/NaCl solution used for reaction clean-ups were 4× up to the adaptor ligation step, and 1.3× after the adaptor ligation step. Size selection of DNA was carried out after the end repair reaction by AMPure XP beads (Beckman Coulter, A63881). The library preparation procedure in brief was as follows. End repair reaction was carried out in 14 μl volume at 20 °C for 30 minutes. Prior to size selection, the volume was increased to 30 μl by adding 16 μl of TE buffer. Amounts of AMPure XP beads used were 0.7× (21 μl) to remove DNA ≥400 bp, and additional 3.3× (99 μl) to collect DNA ≥50 bp, resulting in selecting DNA between sizes of 50 bp and 400 bp. A-tailing reaction was carried out in 10 μl volume at 30 °C for 30 minutes. Adaptor ligation reaction was carried out in 10 μl volume with 10 pmol TruSeq adaptor (for the input samples), or with 1 pmol TruSeq adaptor (for the ChIP samples) at 20 °C for 15 minutes. The ligation product was purified by adding 1.3×amount (13 μl) of PEG/NaCl solution to the reaction, and eluted in 25 μl of Tris-HCl buffer (pH 8.0), followed by another round of purification by adding 1.3×amount (32.4 μl) of PEG/NaCl solution and elution in 11 μl of Tris-HCl buffer (pH 8.0). In order to determine the optimal PCR cycle for the library amplification, qPCR was carried out using KAPA Real-Time Library Amplification Kit (KAPA Biosystems, KK2701) in 10 μl reaction volume with 1.5 μl of the adaptor ligated DNA. 10 μl each of the fluorescence standards 1-4 were used as controls. Threshold PCR cycle that reached the intensity of the fluorescence standard 1 was determined to be the optimal cycle number for library amplification in each sample. Once the PCR cycle was determined, library was amplified in 20 μl reaction volume using KAPA HiFi HotStart ReadyMix (KAPA Biosystems, KK2601) and 8.5 μl of the adaptor ligated DNA. PCR products were purified by adding 1.3× amount (26 μl) of AMPure XP beads to the reaction, and eluted in 20 μl of Tris-HCl buffer (pH 8.0). In order to confirm the quality and quantity of the prepared libraries, their concentration and size distribution were analyzed with Qubit dsDNA HS Assay kit and Agilent High Sensitivity DNA kit. Library qPCR was also carried out to calculate the enrichment folds at positive and negative control regions, using equal amounts of input and ChIP library DNA as templates in the reaction. Enrichment folds were calculated as the ratio of quantified concentrations of "ChIP libraries" and "input libraries". Libraries that displayed enrichment greater than 10 fold at positive control regions were used for sequencing in Illumina HiSeq1500.

a

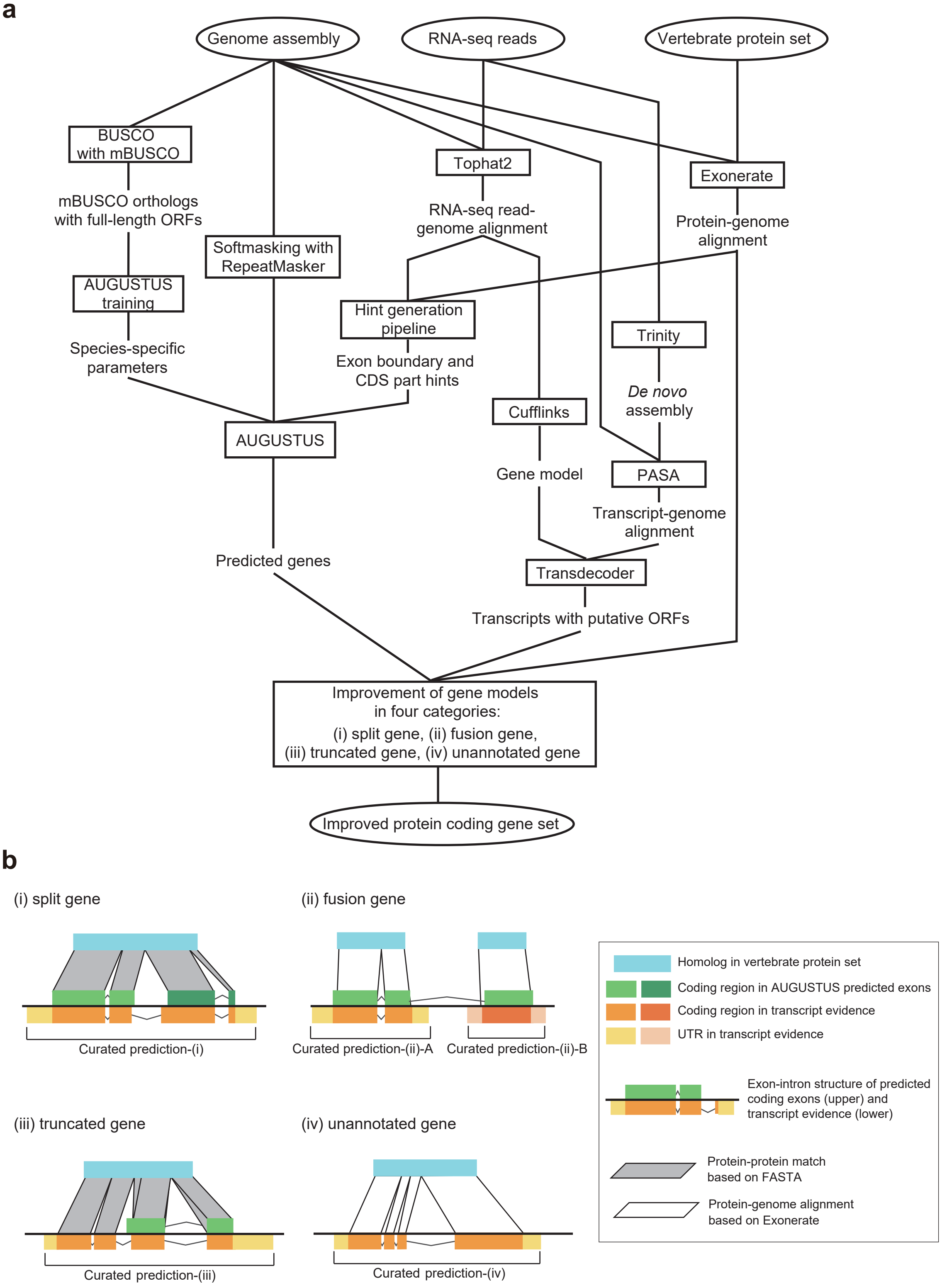

Genome assembly
RNA-seq reads
Vertebrate protein set
BUSCO with mBUSCO
mBUSCO orthologs with full-length ORFs
AUGUSTUS training
Species-specific parameters
Softmasking with RepeatMasker
Tophat2
RNA-seq read-genome alignment
Hint generation pipeline
Exon boundary and CDS part hints
Exonerate
Protein-genome alignment
Trinity
De novo assembly
Cufflinks
Gene model
PASA
Transcript-genome alignment
AUGUSTUS
Predicted genes
Transdecoder
Transcripts with putative ORFs
Improvement of gene models in four categories:
(i) split gene, (ii) fusion gene,
(iii) truncated gene, (iv) unannotated gene
Improved protein coding gene set
(i) split gene
Curated prediction-(i)
(ii) fusion gene
Curated prediction-(ii)-A
Curated prediction-(ii)-B
(iii) truncated gene
Curated prediction-(iii)
(iv) unannotated gene
Curated prediction-(iv)
Homolog in vertebrate protein set
Coding region in AUGUSTUS predicted exons
Coding region in transcript evidence
UTR in transcript evidence
Exon-intron structure of predicted coding exons (upper) and transcript evidence (lower)
Protein-protein match based on FASTA
Protein-genome alignment based on Exonerate

b

Fig. S1. Gene prediction on the *L. camtschaticum* genome assembly. (**a**) A flowchart of gene prediction procedure. Rectangles represent procedures or executed programs. Plain descriptions denote intermediate products. (**b**) Curation of AUGUSTUS prediction with transcript evidence. The four different categories i)-iv) in (**a**), with variable modes of sequence alignment and overlap at genomic position between predicted gene model, transcript evidence, genome assembly, and vertebrate homologs, are schematically shown.

a

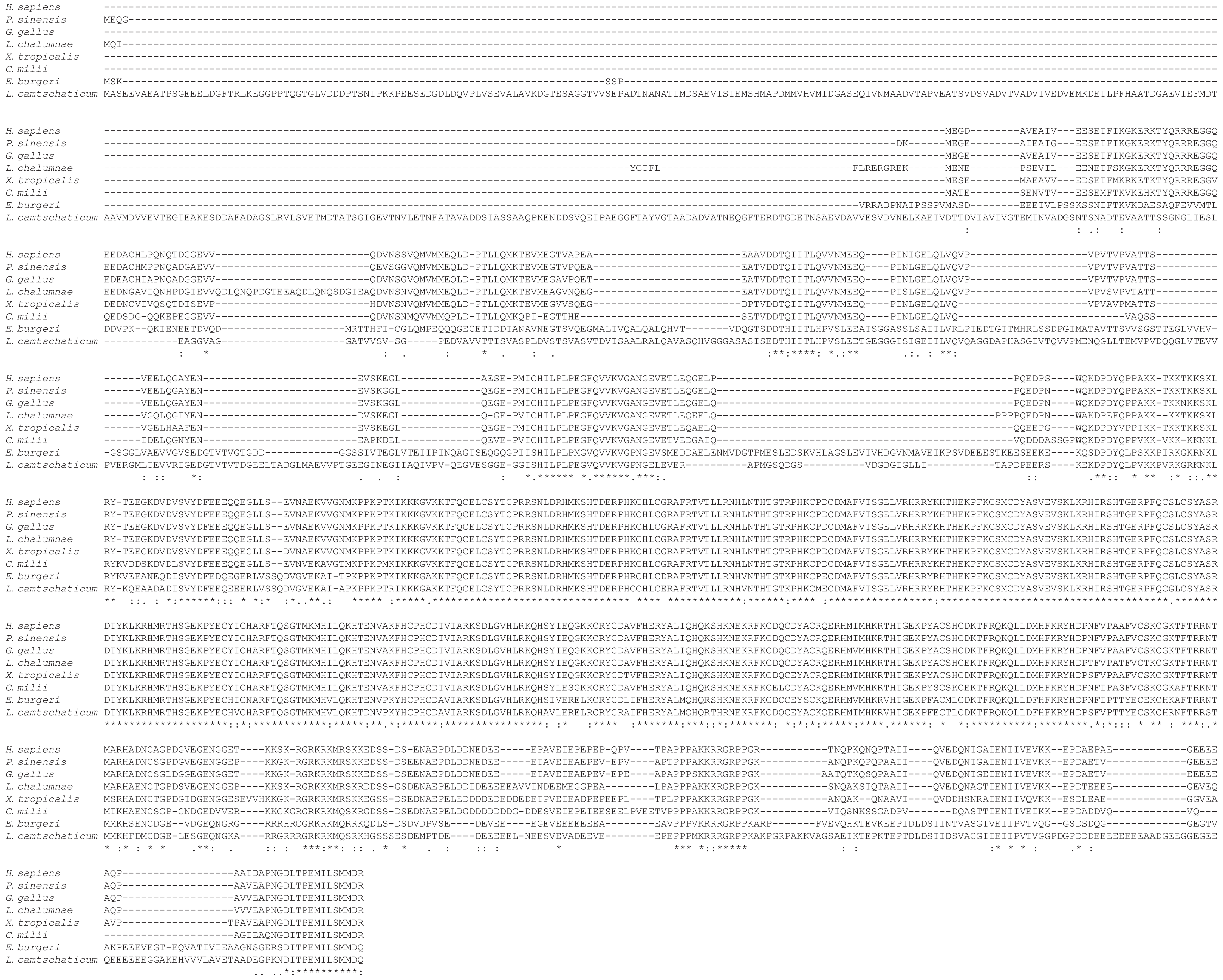

b

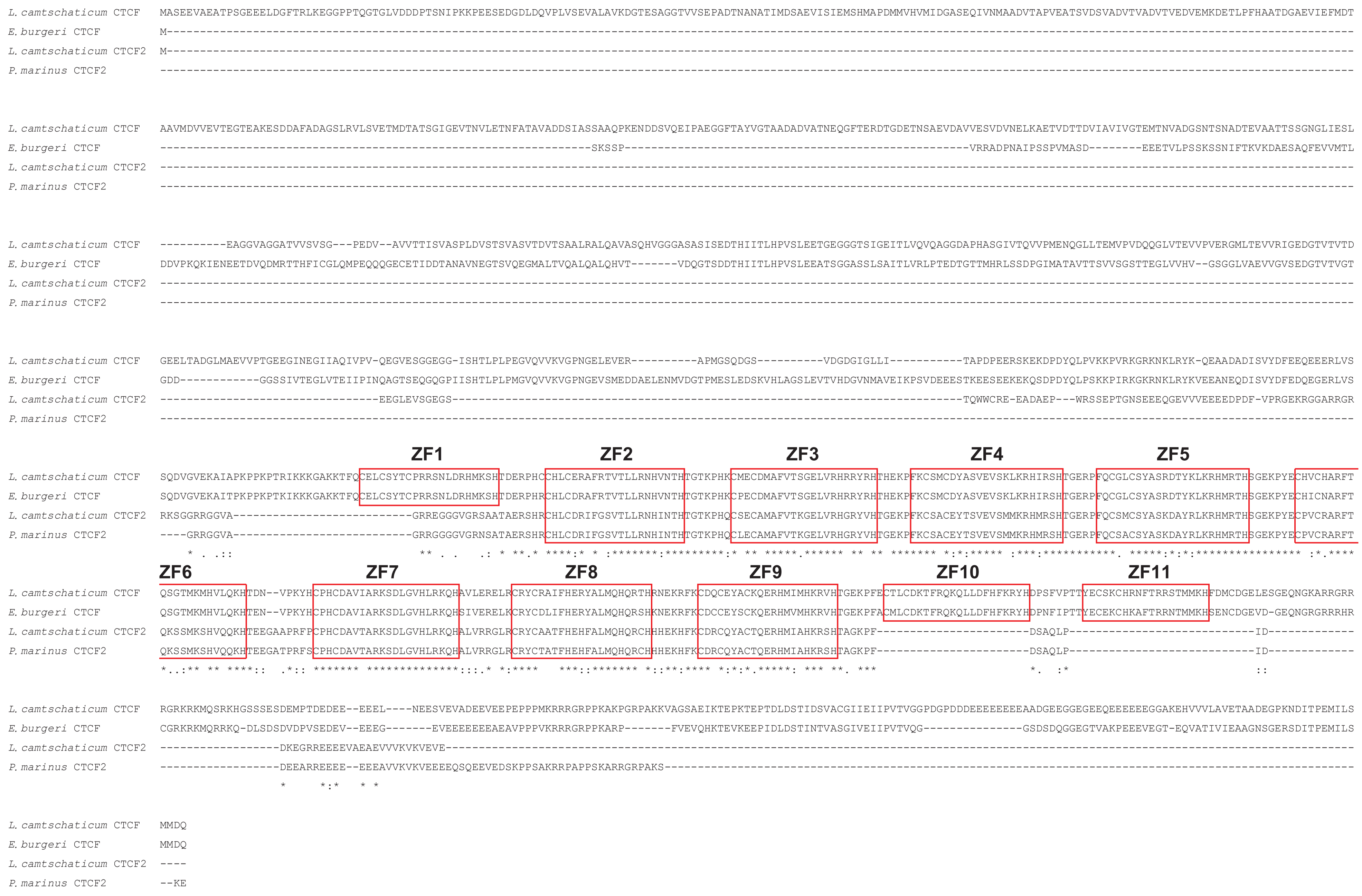

L. camtschaticum CTCF
E. burgeri CTCF
L. camtschaticum CTCF2
P. marinus CTCF2
ZF1
ZF2
ZF3
ZF4
ZF5
ZF6
ZF7
ZF8
ZF9
ZF10
ZF11

Fig. S2. Alignment of vertebrate CTCF peptide sequences by MAFFT program. Alignments were performed by MAFFT program (v7.293, http://mafft.cbrc.jp/alignment/software/) by iterative refinement method (L-INS-i). (**a**) Alignment of LjCTCF and vertebrate CTCF peptides. (**b**) Alignment of cyclostome CTCF and CTCF2 peptides. Red boxes indicate conserved Zn finger (ZF) domains inferred by MOTIF Search program (http://www.genome.jp/tools/motif/) that matched Pfam and PROSITE patterns.

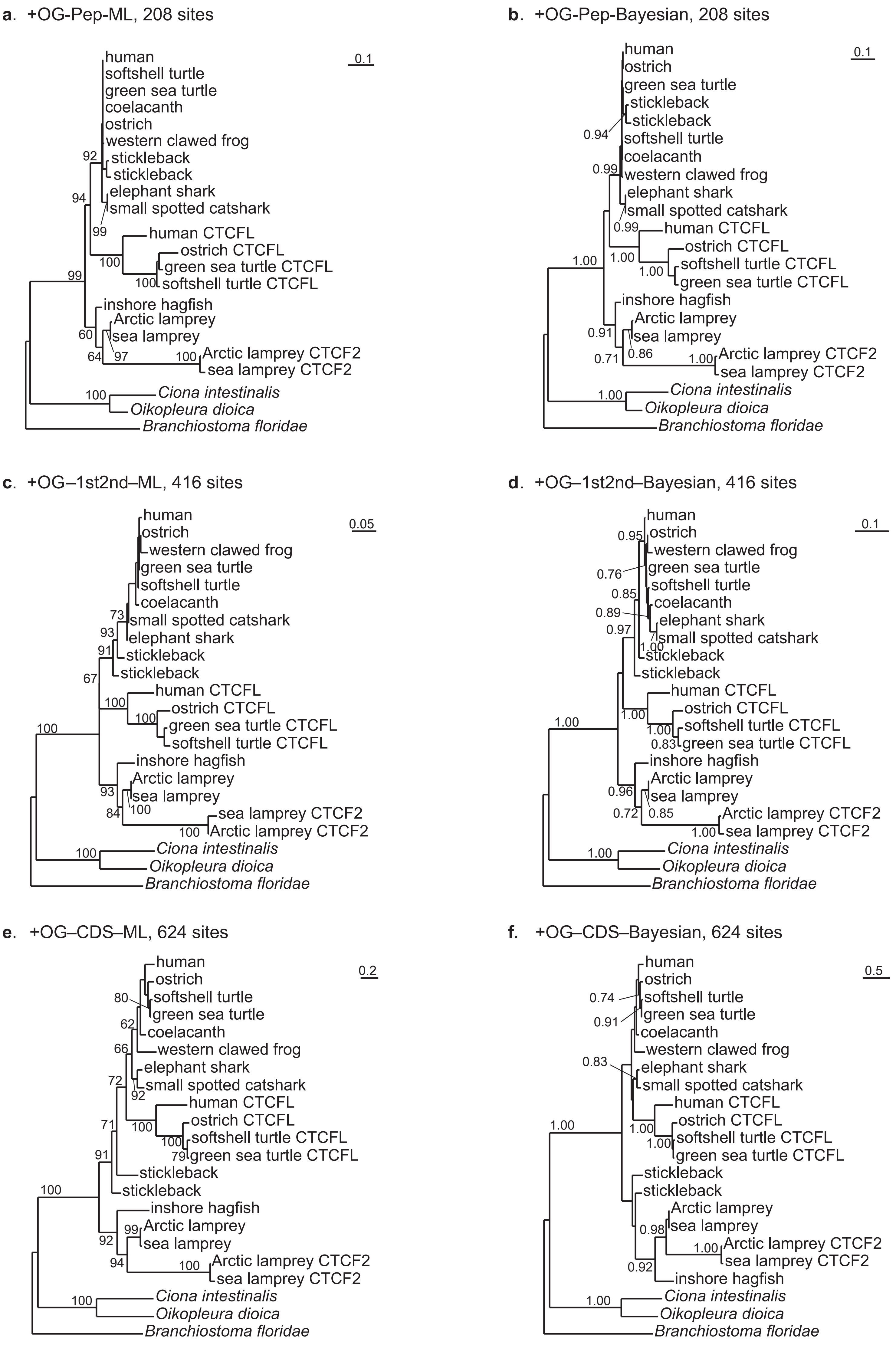
a. +OG-Pep-ML, 208 sites
0.1
human
softshell turtle
green sea turtle
coelacanth
ostrich
western clawed frog
stickleback
stickleback
elephant shark
small spotted catshark
human CTCFL
ostrich CTCFL
green sea turtle CTCFL
softshell turtle CTCFL
inshore hagfish
Arctic lamprey
sea lamprey
Arctic lamprey CTCF2
sea lamprey CTCF2
Ciona intestinalis
Oikopleura dioica
Branchiostoma floridae
92
94
99
100
100
99
60
64
97
100
100
b. +OG-Pep-Bayesian, 208 sites
0.1
human
ostrich
green sea turtle
stickleback
stickleback
softshell turtle
coelacanth
western clawed frog
elephant shark
small spotted catshark
human CTCFL
ostrich CTCFL
softshell turtle CTCFL
green sea turtle CTCFL
inshore hagfish
Arctic lamprey
sea lamprey
Arctic lamprey CTCF2
sea lamprey CTCF2
Ciona intestinalis
Oikopleura dioica
Branchiostoma floridae
0.94
0.99
0.99
1.00
1.00
1.00
0.91
0.71
0.86
1.00
1.00
c. +OG–1st2nd–ML, 416 sites
0.05
human
ostrich
western clawed frog
green sea turtle
softshell turtle
coelacanth
small spotted catshark
elephant shark
stickleback
stickleback
human CTCFL
ostrich CTCFL
green sea turtle CTCFL
softshell turtle CTCFL
inshore hagfish
Arctic lamprey
sea lamprey
sea lamprey CTCF2
Arctic lamprey CTCF2
Ciona intestinalis
Oikopleura dioica
Branchiostoma floridae
73
93
91
67
100
100
100
93
84
100
100
100
d. +OG–1st2nd–Bayesian, 416 sites
0.1
human
ostrich
western clawed frog
green sea turtle
softshell turtle
coelacanth
elephant shark
small spotted catshark
stickleback
stickleback
human CTCFL
ostrich CTCFL
softshell turtle CTCFL
green sea turtle CTCFL
inshore hagfish
Arctic lamprey
sea lamprey
Arctic lamprey CTCF2
sea lamprey CTCF2
Ciona intestinalis
Oikopleura dioica
Branchiostoma floridae
0.95
0.76
0.85
0.89
0.97
1.00
1.00
1.00
1.00
0.83
0.96
0.72
0.85
1.00
1.00
e. +OG–CDS–ML, 624 sites
0.2
human
ostrich
softshell turtle
green sea turtle
coelacanth
western clawed frog
elephant shark
small spotted catshark
human CTCFL
ostrich CTCFL
softshell turtle CTCFL
green sea turtle CTCFL
stickleback
stickleback
inshore hagfish
Arctic lamprey
sea lamprey
Arctic lamprey CTCF2
sea lamprey CTCF2
Ciona intestinalis
Oikopleura dioica
Branchiostoma floridae
80
62
66
72
92
71
100
100
79
91
100
99
92
94
100
100
f. +OG–CDS–Bayesian, 624 sites
0.5
human
ostrich
softshell turtle
green sea turtle
coelacanth
western clawed frog
elephant shark
small spotted catshark
human CTCFL
ostrich CTCFL
softshell turtle CTCFL
green sea turtle CTCFL
stickleback
stickleback
Arctic lamprey
sea lamprey
Arctic lamprey CTCF2
sea lamprey CTCF2
inshore hagfish
Ciona intestinalis
Oikopleura dioica
Branchiostoma floridae
0.74
0.91
0.83
1.00
1.00
1.00
0.98
1.00
0.92
1.00

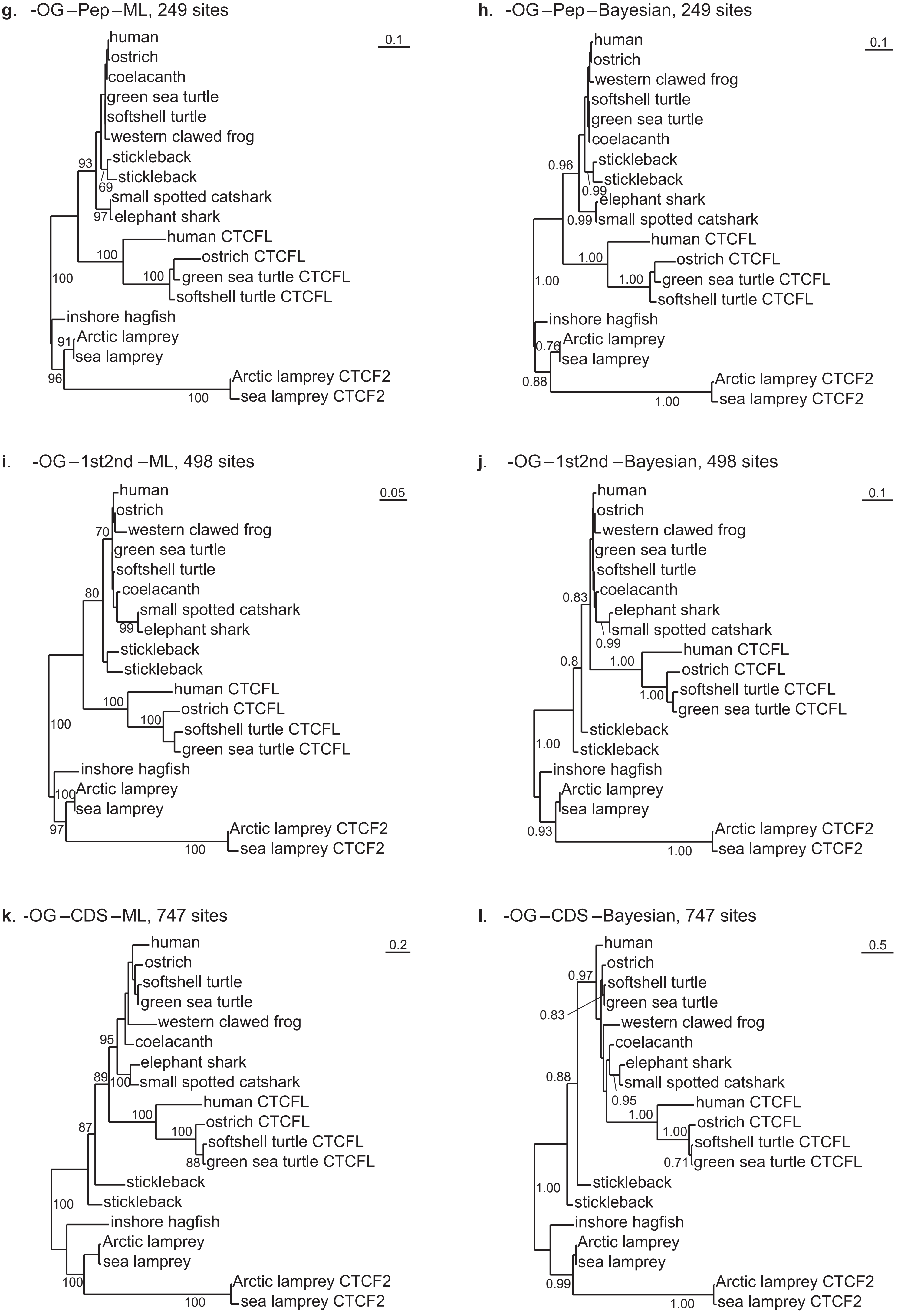
g. -OG –Pep –ML, 249 sites
0.1
human
ostrich
coelacanth
green sea turtle
softshell turtle
western clawed frog
stickleback
stickleback
small spotted catshark
elephant shark
human CTCFL
ostrich CTCFL
green sea turtle CTCFL
softshell turtle CTCFL
inshore hagfish
Arctic lamprey
sea lamprey
Arctic lamprey CTCF2
sea lamprey CTCF2
93
69
97
100
100
100
91
96
100
h. -OG –Pep –Bayesian, 249 sites
0.1
human
ostrich
western clawed frog
softshell turtle
green sea turtle
coelacanth
stickleback
stickleback
elephant shark
small spotted catshark
human CTCFL
ostrich CTCFL
green sea turtle CTCFL
softshell turtle CTCFL
inshore hagfish
Arctic lamprey
sea lamprey
Arctic lamprey CTCF2
sea lamprey CTCF2
0.96
0.99
0.99
1.00
1.00
1.00
0.76
0.88
1.00
i. -OG –1st2nd –ML, 498 sites
0.05
human
ostrich
western clawed frog
green sea turtle
softshell turtle
coelacanth
small spotted catshark
elephant shark
stickleback
stickleback
human CTCFL
ostrich CTCFL
softshell turtle CTCFL
green sea turtle CTCFL
inshore hagfish
Arctic lamprey
sea lamprey
Arctic lamprey CTCF2
sea lamprey CTCF2
70
80
99
100
100
100
100
97
100
j. -OG –1st2nd –Bayesian, 498 sites
0.1
human
ostrich
western clawed frog
green sea turtle
softshell turtle
coelacanth
elephant shark
small spotted catshark
human CTCFL
ostrich CTCFL
softshell turtle CTCFL
green sea turtle CTCFL
stickleback
stickleback
inshore hagfish
Arctic lamprey
sea lamprey
Arctic lamprey CTCF2
sea lamprey CTCF2
0.83
0.99
0.8
1.00
1.00
1.00
0.93
1.00
k. -OG –CDS –ML, 747 sites
0.2
human
ostrich
softshell turtle
green sea turtle
western clawed frog
coelacanth
elephant shark
small spotted catshark
human CTCFL
ostrich CTCFL
softshell turtle CTCFL
green sea turtle CTCFL
stickleback
stickleback
inshore hagfish
Arctic lamprey
sea lamprey
Arctic lamprey CTCF2
sea lamprey CTCF2
95
89
100
100
100
88
87
100
100
100
l. -OG –CDS –Bayesian, 747 sites
0.5
human
ostrich
softshell turtle
green sea turtle
western clawed frog
coelacanth
elephant shark
small spotted catshark
human CTCFL
ostrich CTCFL
softshell turtle CTCFL
green sea turtle CTCFL
stickleback
stickleback
inshore hagfish
Arctic lamprey
sea lamprey
Arctic lamprey CTCF2
sea lamprey CTCF2
0.97
0.83
0.88
0.95
1.00
1.00
0.71
1.00
0.99
1.00

Fig. S3. CTCF molecular phylogeny inferred with variable methods and datasets. Each of the trees were inferred with a combination of the three conditions, namely 1) inclusion/exclusion of an outgroup (+OG or -OG), 2) type of input sequences [amino acids (Pep), the first and second codon positions (1st2nd), or entire coding regions (CDS)], and 3) choice of the tree inference method (ML or Bayesian). (**a**) Phylogenetic tree with an outgroup inferred using the amino acid sequences with the ML approach (designated '+OG–Pep–ML' using the abbreviations above). The conditions of the other trees are abbreviated as follows: (**b**) -OG–Pep–Bayesian; (**c**) +OG–1st2nd–ML; (**d**) +OG–1st2nd–Bayesian; (**e**) +OG–CDS–ML; (**f**) +OG–CDS–Bayesian; (**g**) -OG–Pep–ML; (**h**) -OG–Pep–Bayesian; (**i**) -OG–1st2nd–ML; (**f**) -OG–1st2nd–Bayesian; (**k**) -OG–CDS–ML; (**l**) -OG–CDS–Bayesian. Two stickleback sequences for duplicated genes are included (upper, Ensembl ENSGACP00000003270; lower, ENSGACP00000020939). For ML tree inference, the PROTCATWAG and CATGTR models were employed for amino acid and nucleotide sequences, respectively, and for Bayesian tree inference, the CAT-WAG-Γ and CAT-GTR-Γ models were employed. Bootstrap values of 60 or greater and posterior probabilities of 0.7 or greater were shown at the branch nodes of the ML and Bayesian trees, respectively.

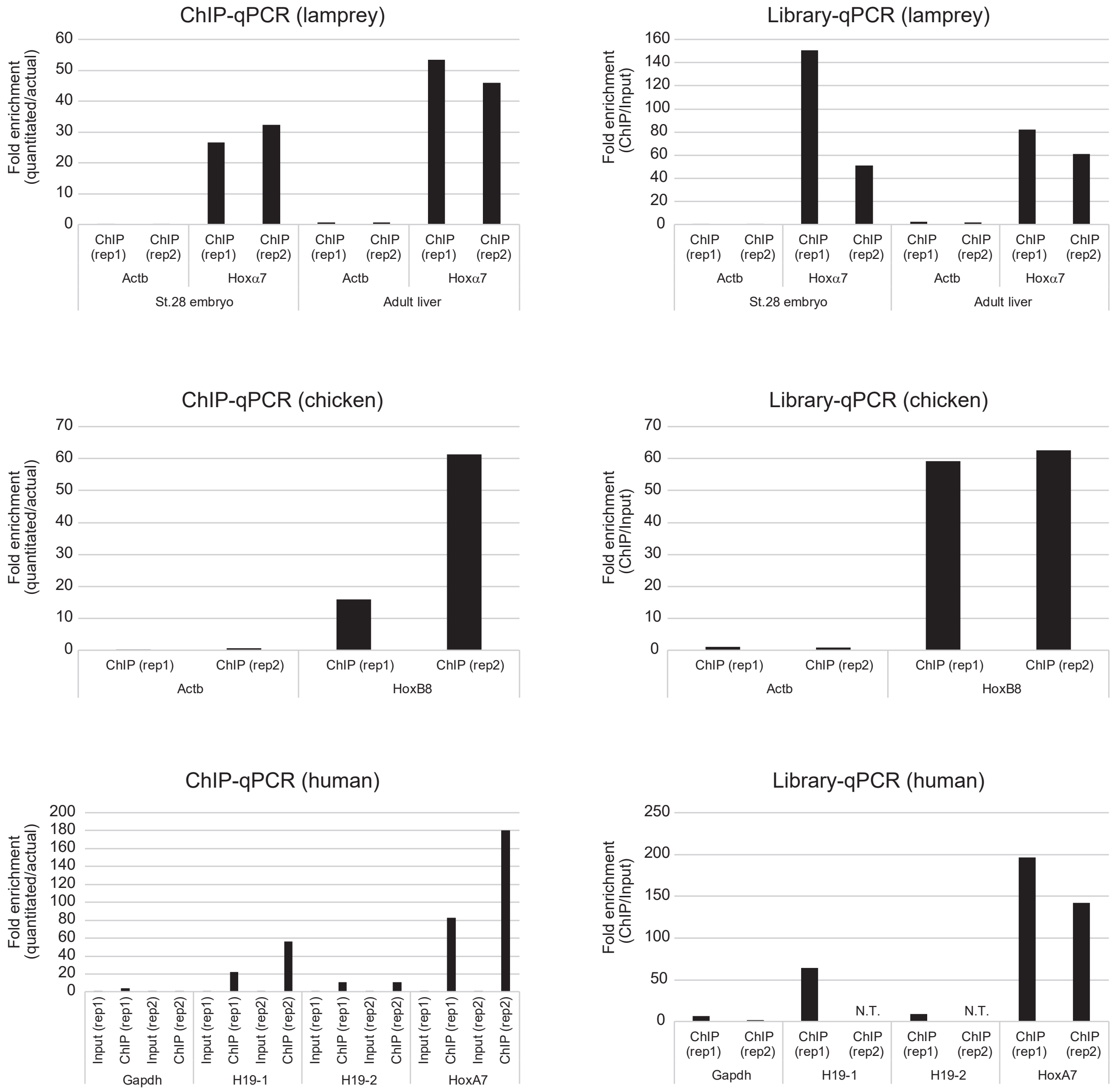

Fig. S4. Enrichment analysis of CTCF bound regions. qPCR was performed using QuantiTect SYBR Green PCR kit (Qiagen) with primers described in Supplementary Table S7. Template DNAs used for qPCR were input and ChIP DNAs for the ChIP-qPCR experiment (on the left), or the input and ChIP DNAlibraries for the library-qPCR experiment (on the right). For ChIP-qPCR, enrichment folds were calculated as the ratio between the "qPCR-quantitated concentration" and "actual DNA concentration" . For library-qPCR, equal amounts of template DNAs were used for the qPCR reaction, and enrichment folds were calculated as the ratio of quantitated values of "ChIP library" and "input library" . ChIP libraries were confirmed to display no less than 10 folds of enrichment in at least one positive control region before sequencing.

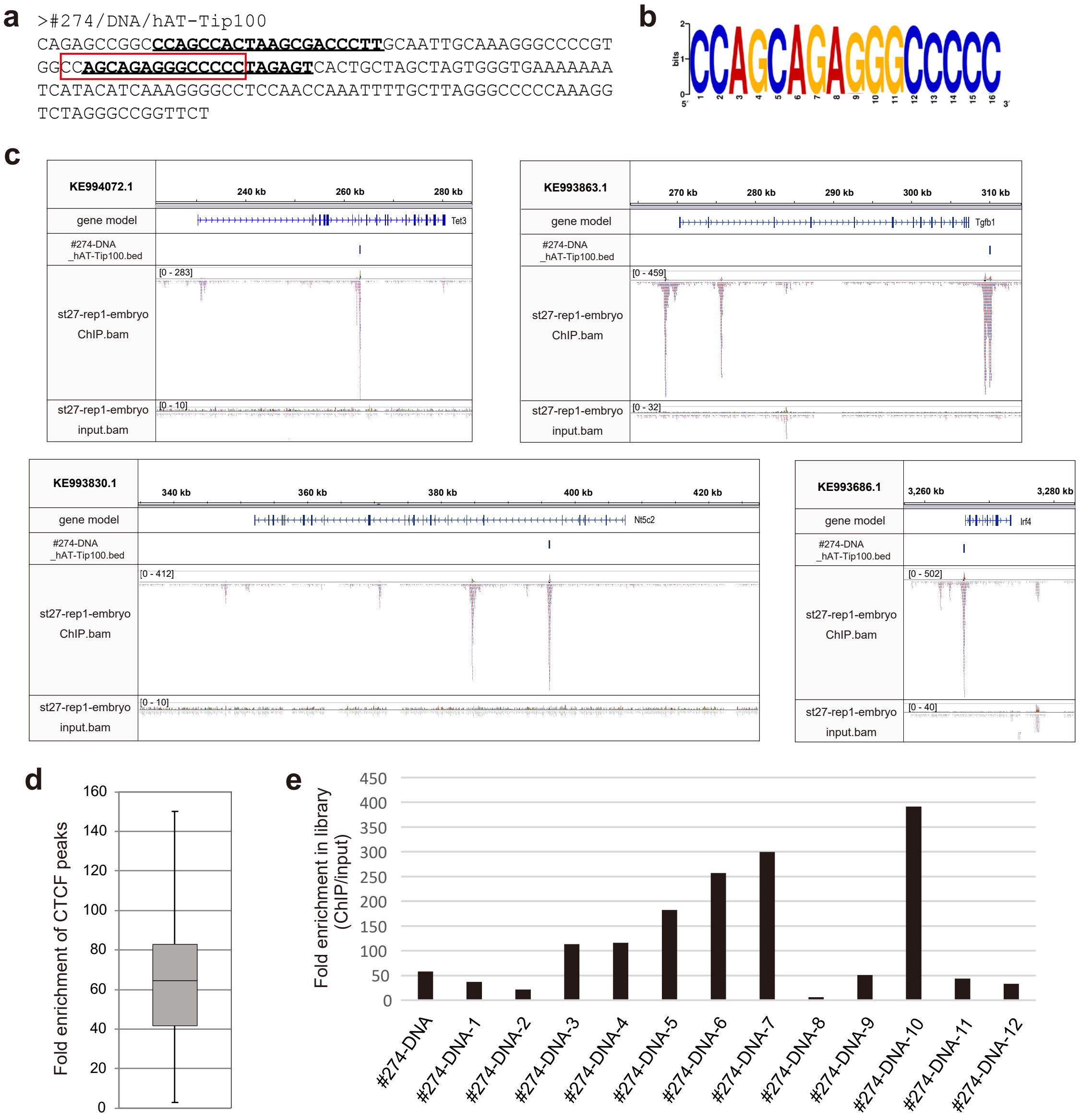

Fig. S5. Association of CTCF binding sites with the hAT-Tip100 DNA transposable element in Arctic lamprey. (**a**) Consensus sequence of the repeat #274/DNA/hAT-Tip100 identified by RepeatModeler. Potential CTCF binding sequence is highlighted in the red box. Positions of the primers used for ChIP qPCR (#274-DNA-f/r) are shown in bold with underlines. (**b**) Motif logo of the CTCF binding sequence. FIMO program identified putative CTCF binding sequences in 504 out of 586 #274/DNA/hAT-Tip100 repeat regions in the LetJap1.0 genome. Putative CTCF binding sequences analyzed by WebLogo (http://weblogo.berkeley.edu/) showed a high level of similarity among the identified 504 regions. (**c**) CTCF ChIP-seq peaks that overlap with #274/DNA/hAT-Tip100 repeats. CTCF ChIP-seq peaks in the stage 27 embryos overlap with the #274/DNA/hAT-Tip100 repeat in *Tet3*, *Tgfb1*, *Nt5c2*, and *Irf4* gene regions. (**d**) Fold enrichment distribution of ChIP-seq peaks that overlap with #274/DNA/hAT-Tip100 repeat. Fold-enrichment values of 512 MACS2 identified peaks were analyzed. Box and the inner horizontal line represents the first and third quartile and the median; bar ends represent the minimum and maximum fold enrichment values. (**e**) qPCR validation was carried out using input library DNA and ChIP library DNA as templates. Primers used were designed within the consensus sequence of #274/DNA/hAT-Tip100 repeat and also for twelve selected regions (Supplementary Table S7). Shown are the fold enrichment values of ChIP samples against the input sample.

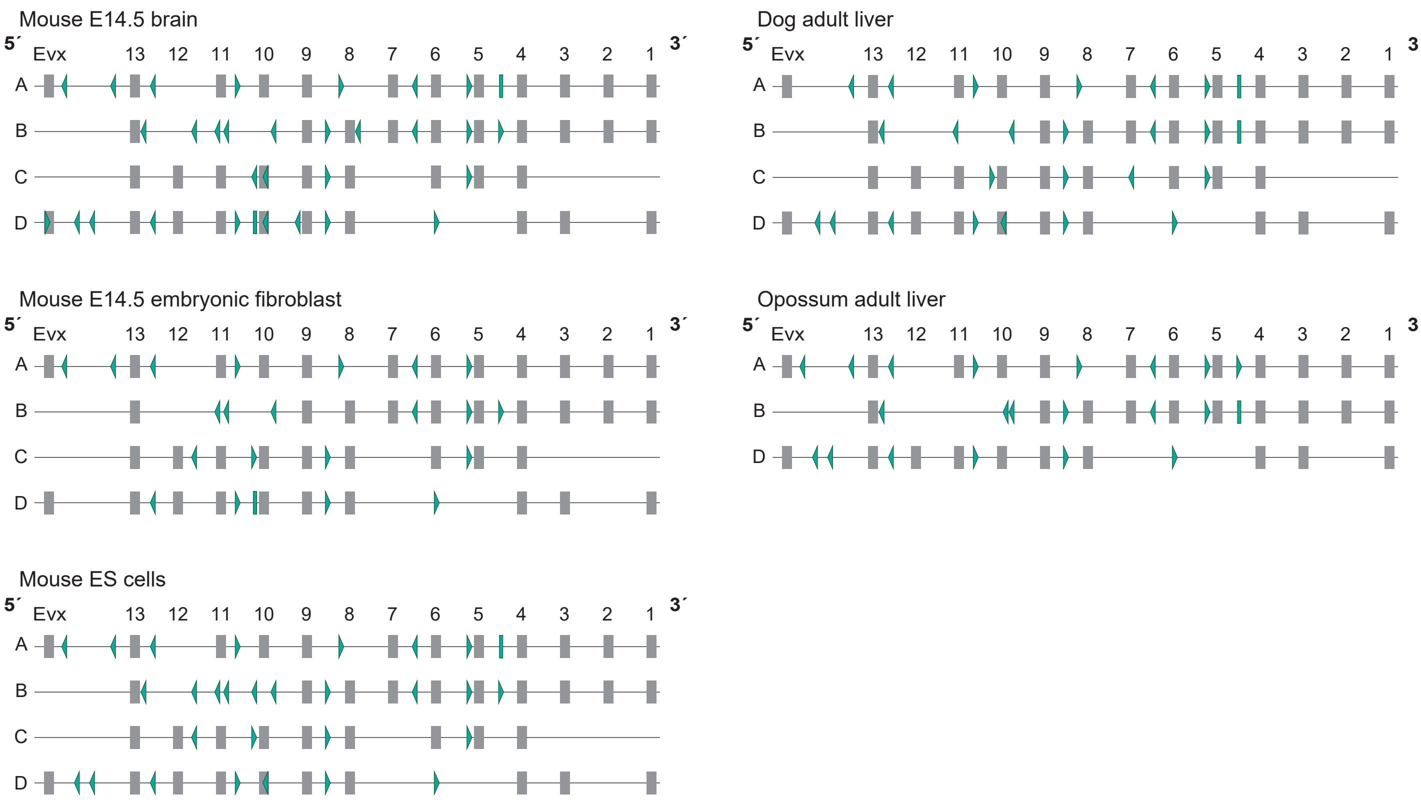

Fig. S6. Distribution of CTCF binding sites at Hox clusters of mouse E14.5 embryonic tissues (brain and MEF), mouse ES cells, adult dog liver, and adult opossum liver. ChIP-seq data were obtained from NCBI SRA (SRR392354, SRR505014, SRR207080, SRR207071, SRR207089, and SRR207081) and EBI ENA (ERR022285, ERR022304, ERR022303, ERR022307, ERR022306, and ERR022301) and processed as described in Supplementary Materials and Methods. Coding regions of genes are indicated with gray boxes. CTCF binding sites are indicated with green arrowheads and bars. Arrowheads indicate the orientations of core motifs inferred by the FIMO program. Bars indicate CTCF binding sites without a core motif. ChIP-seq peaks with enrichments of no less than 10 fold (for mouse and dog), or 5 fold (for opossum) are shown. Note that Hox C genes were not identified as a cluster in the opossum genome assembly MonDom5.

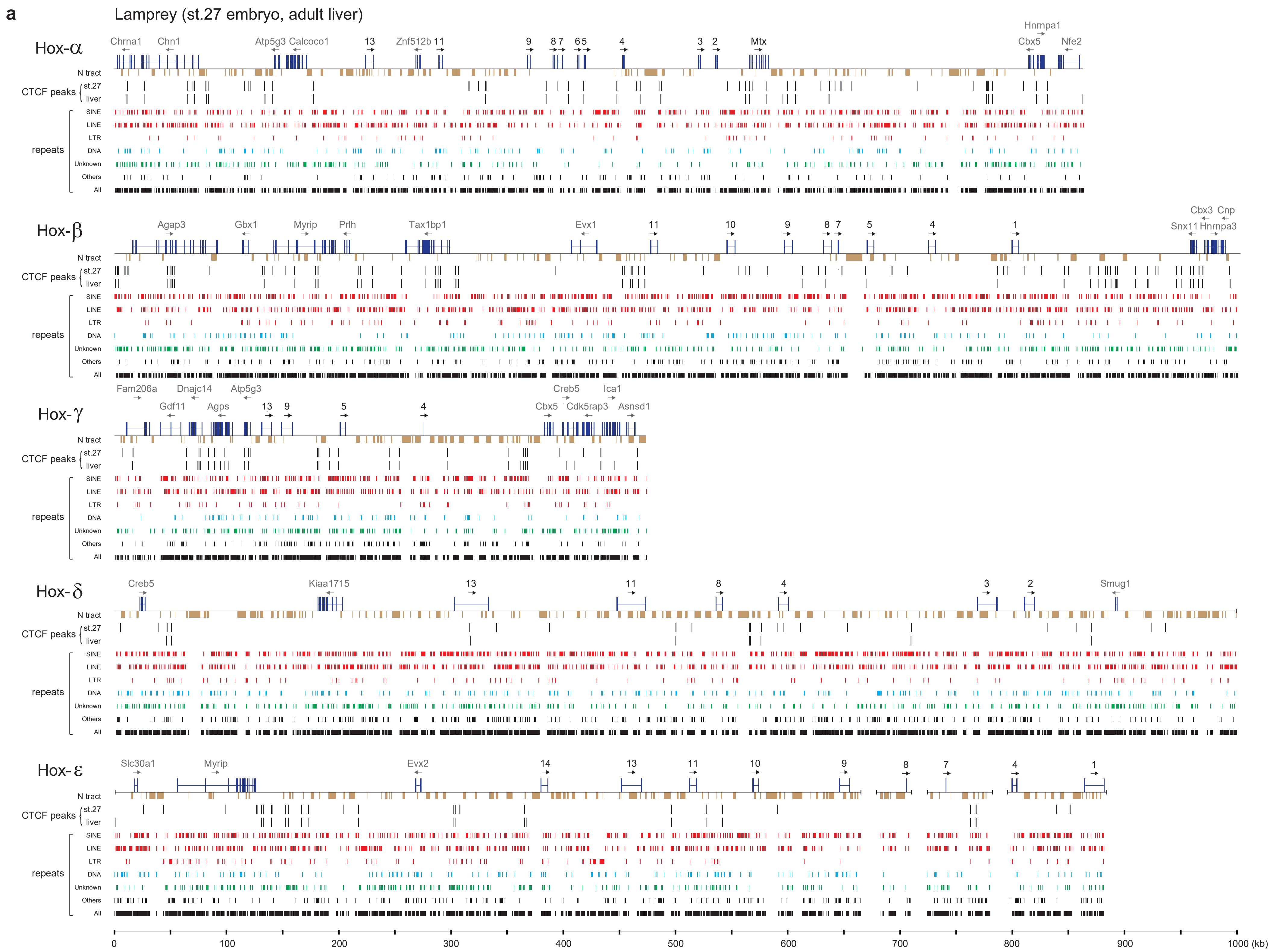

a
Lamprey (st.27 embryo, adult liver)
Hox-α
Hox-β
Hox-γ
Hox-δ
Hox-ε
N tract
CTCF peaks
st.27
liver
repeats
SINE
LINE
LTR
DNA
Unknown
Others
All
Chrna1
Chn1
Atp5g3
Calcoco1
Znf512b
Mtx
Hnrnpa1
Cbx5
Nfe2
Agap3
Gbx1
Myrip
Prlh
Tax1bp1
Evx1
Cbx3
Cnp
Snx11
Hnrnpa3
Fam206a
Dnajc14
Atp5g3
Gdf11
Agps
Creb5
Ica1
Cbx5
Cdk5rap3
Asnsd1
Creb5
Kiaa1715
Smug1
Slc30a1
Myrip
Evx2
0
100
200
300
400
500
600
700
800
900
1000 (kb)

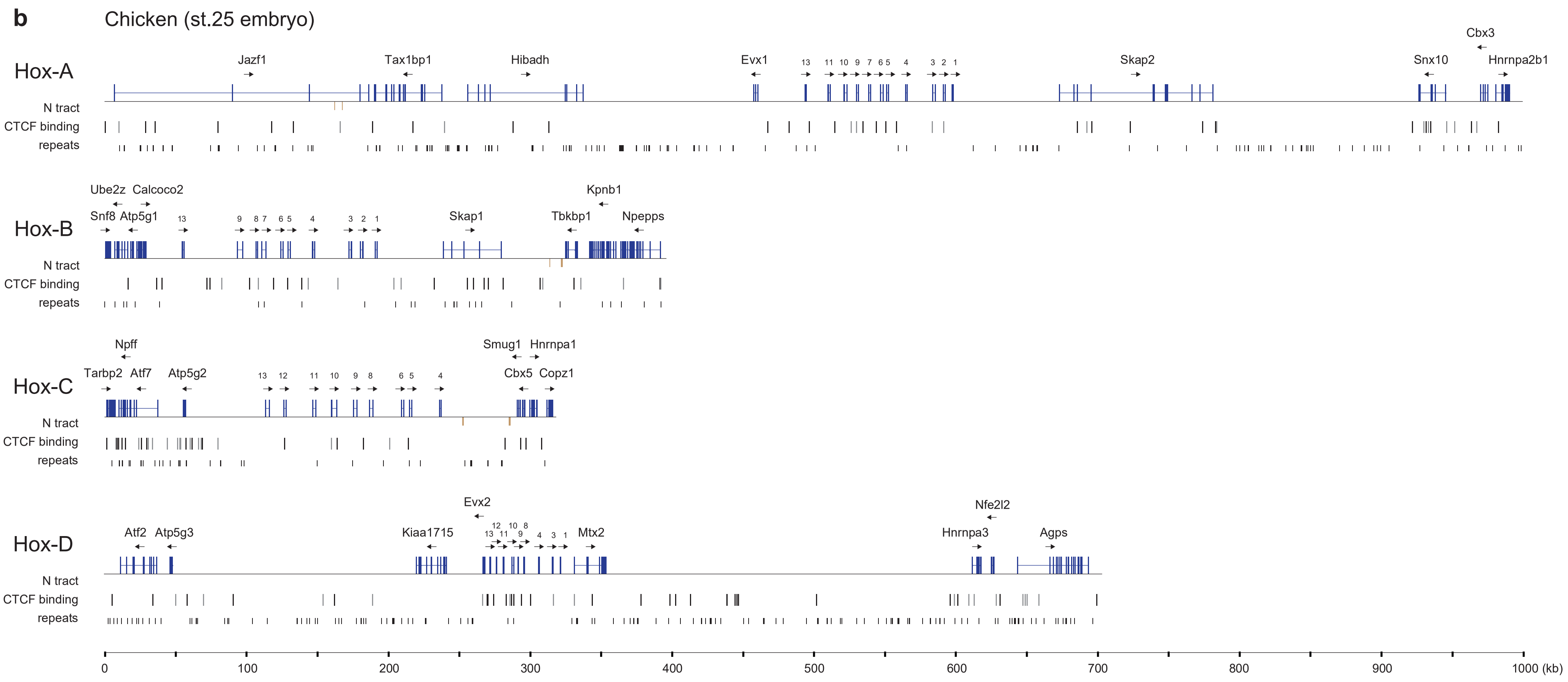
b
Chicken (st.25 embryo)
Hox-A
N tract
CTCF binding
repeats
Jazf1
Tax1bp1
Hibadh
Evx1
13 11 10 9 7 6 5 4 3 2 1
Skap2
Snx10
Cbx3
Hnrnpa2b1
Hox-B
Ube2z
Calcoco2
Snf8
Atp5g1
13 9 8 7 6 5 4 3 2 1
Skap1
Kpnb1
Tbkbp1
Npepps
Hox-C
Npff
Tarbp2
Atf7
Atp5g2
13 12 11 10 9 8 6 5 4
Smug1
Hnrnpa1
Cbx5
Copz1
Hox-D
Atf2
Atp5g3
Kiaa1715
Evx2
13 12 11 10 9 8 4 3 1
Mtx2
Nfe2l2
Hnrnpa3
Agps
0 100 200 300 400 500 600 700 800 900 1000 (kb)

c

Mouse (E14.5 brain, E14.5 MEF, ES cells)

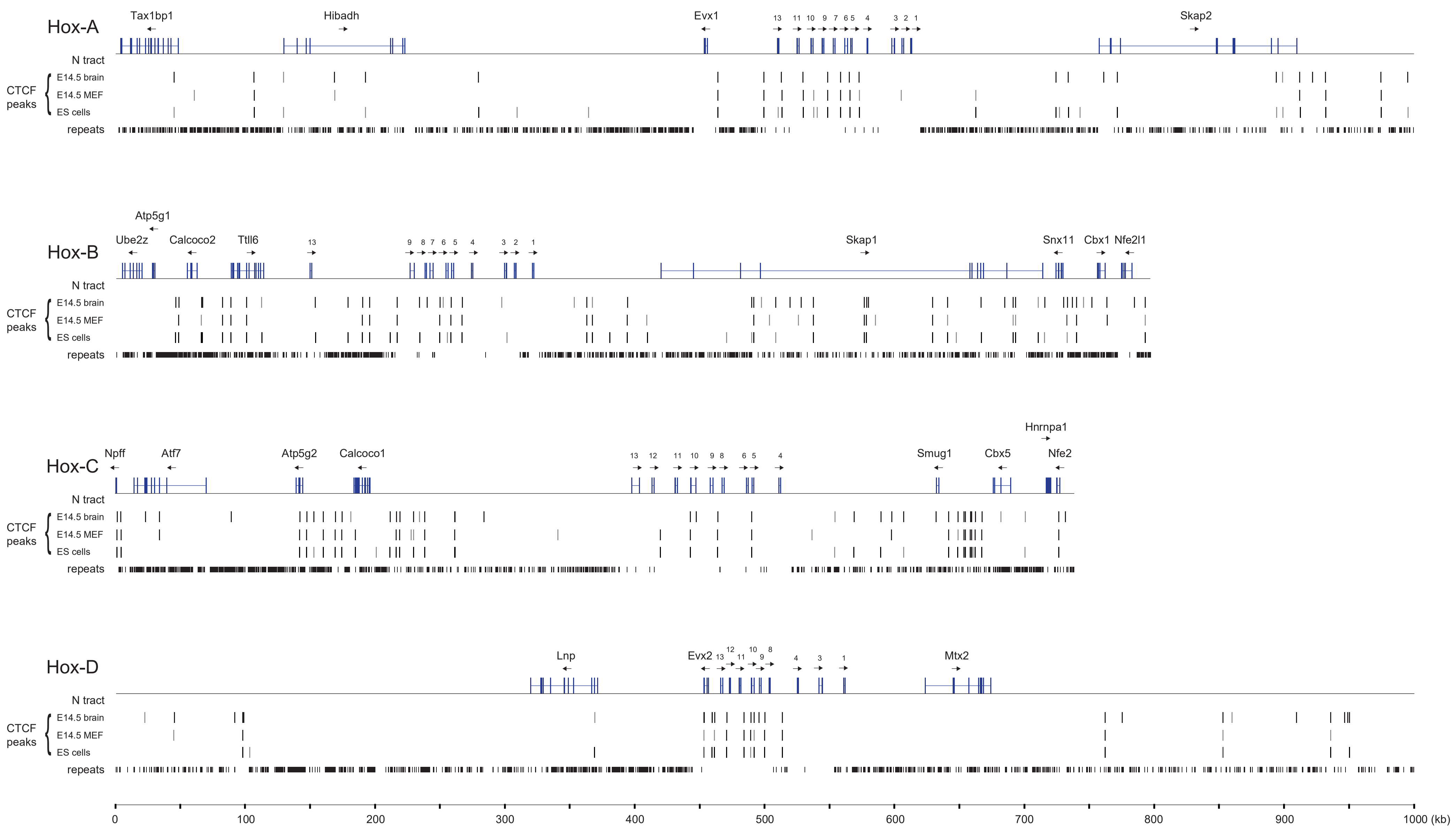

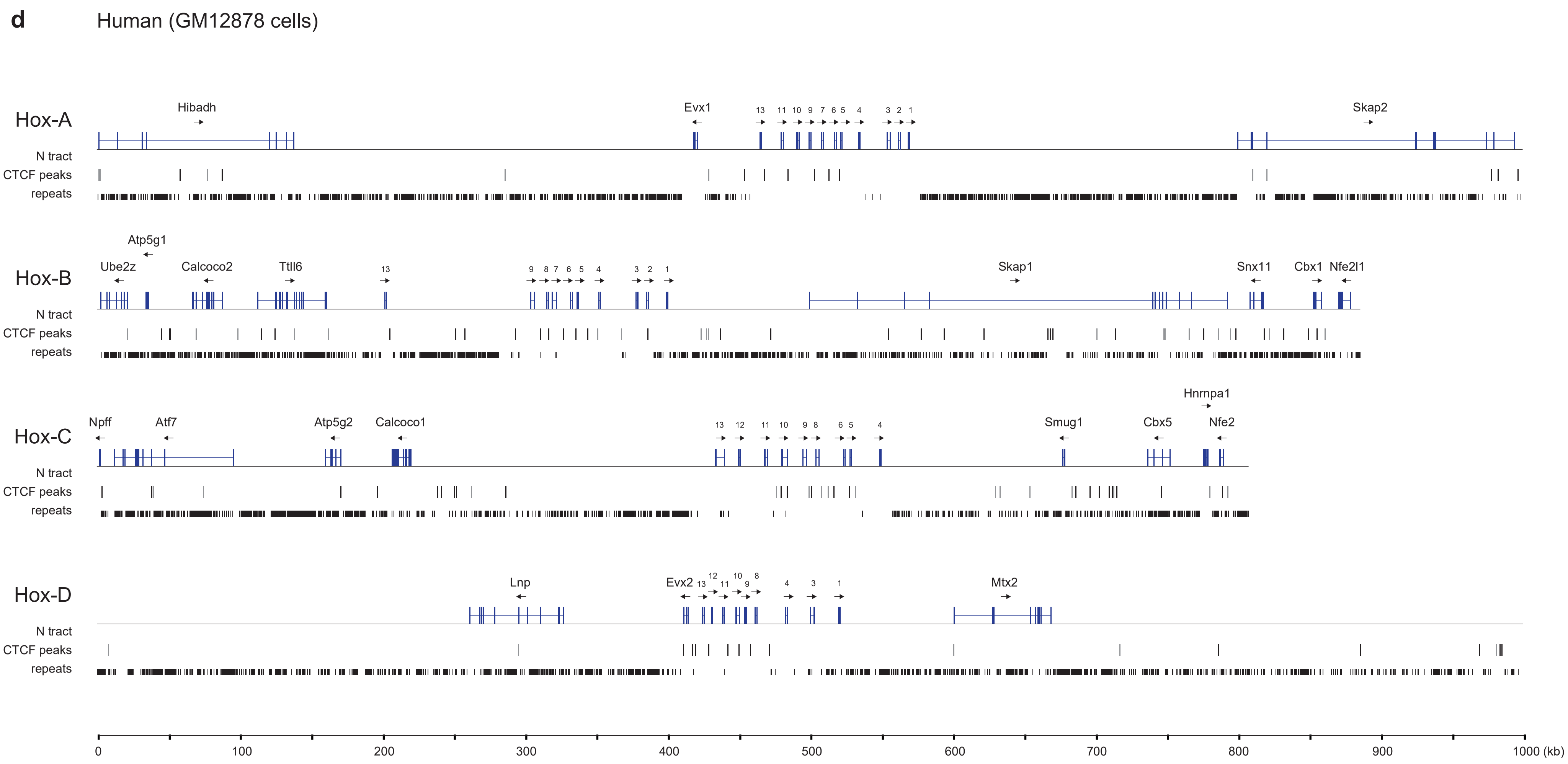

d
Human (GM12878 cells)
Hox-A
Hibadh
Evx1
13 11 10 9 7 6 5 4 3 2 1
Skap2
N tract
CTCF peaks
repeats
Hox-B
Atp5g1
Ube2z
Calcoco2
Ttll6
13
9 8 7 6 5 4 3 2 1
Skap1
Snx11
Cbx1
Nfe2l1
Hox-C
Npff
Atf7
Atp5g2
Calcoco1
13 12 11 10 9 8 6 5 4
Smug1
Cbx5
Hnrnpa1
Nfe2
Hox-D
Lnp
Evx2
13 12 11 10 9 8 4 3 1
Mtx2
0
100
200
300
400
500
600
700
800
900
1000 (kb)

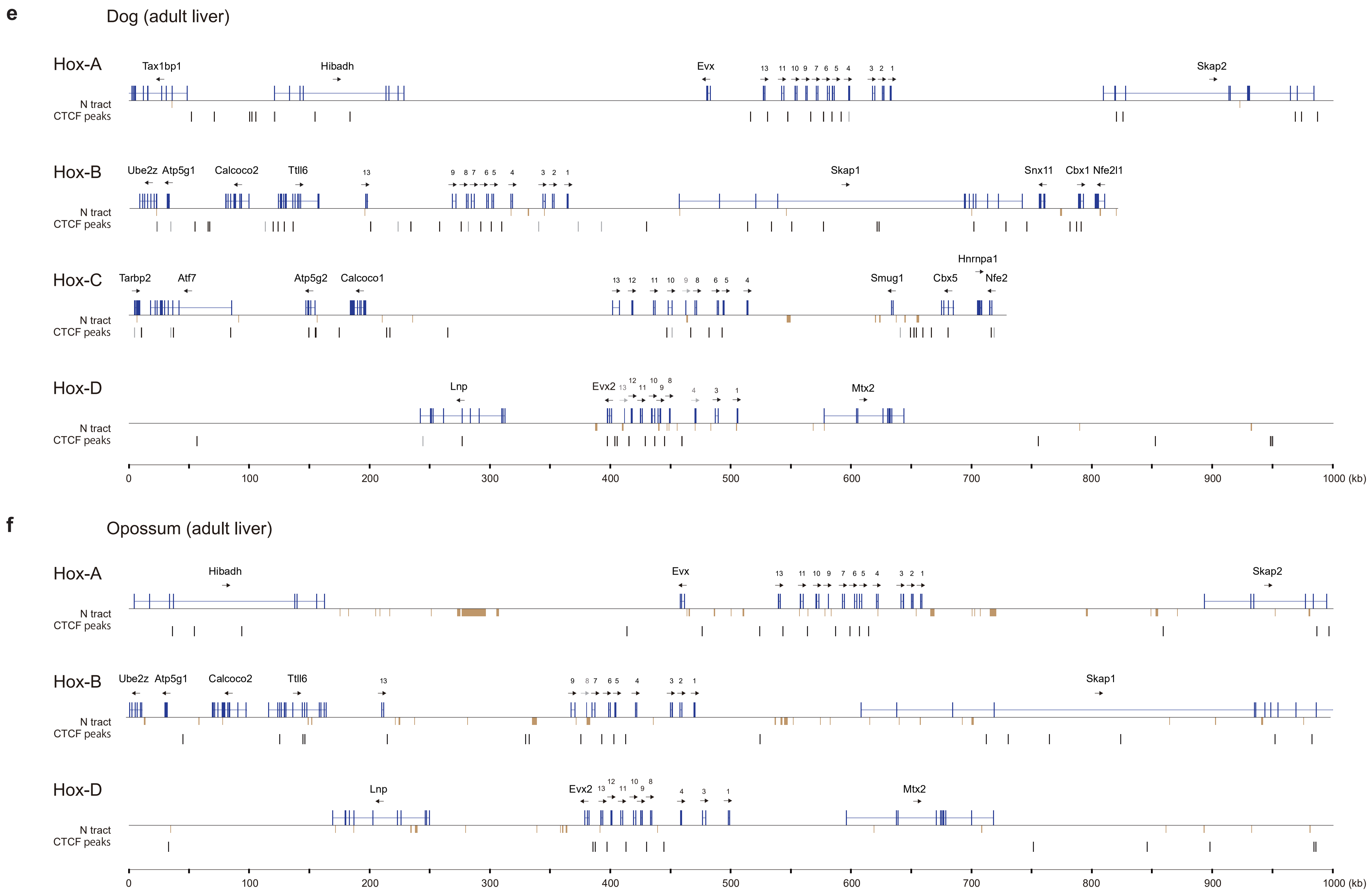
e
Dog (adult liver)
Hox-A
Tax1bp1
Hibadh
Evx
13 11 10 9 7 6 5 4 3 2 1
Skap2
N tract
CTCF peaks
Hox-B
Ube2z Atp5g1
Calcoco2
Ttll6
13
9 8 7 6 5 4 3 2 1
Skap1
Snx11
Cbx1 Nfe2l1
Hox-C
Tarbp2
Atf7
Atp5g2
Calcoco1
13 12 11 10 9 8 6 5 4
Smug1
Cbx5
Hnrnpa1
Nfe2
Hox-D
Lnp
Evx2 13 12 11 10 9 8 4 3 1
Mtx2
0 100 200 300 400 500 600 700 800 900 1000 (kb)
f
Opossum (adult liver)
Hox-A
Hibadh
Evx
13 11 10 9 7 6 5 4 3 2 1
Skap2
Hox-B
Ube2z Atp5g1
Calcoco2
Ttll6
13
9 8 7 6 5 4 3 2 1
Skap1
Hox-D
Lnp
Evx2 13 12 11 10 9 8 4 3 1
Mtx2
0 100 200 300 400 500 600 700 800 900 1000 (kb)

Fig. S7. Lamprey, chicken, mouse, human, dog, and opossum Hox gene clusters with locations of CTCF ChIP-seq peaks. (**a**) Lamprey Hox gene clusters in the LetJap1.0 genome assembly. α (on scaffold KE993675.1), β (on scaffold KE993672.1), γ (on scaffold KE993746.1), δ (on scaffold KE993858.1), and ε (on scaffolds KE993970.1, KE995814.1, KE995158.1, and KE994851.1) clusters are shown. Gene names given by Mehta *et al.*[2] are indicated in black letters. Gene names in grey letters are the names of human RefSeq proteins that showed the highest similarity in BLASTP searches in this study. CTCF peaks identified in stage 27 embryo, and adult liver samples are shown. Included in 'Others' are the repeats (52 entries) classified as RC/Helitron, satellite, simple repeat, buffer, and rRNA. (**b**) Chicken Hox gene clusters in the galGal5 genome assembly. CTCF peaks in stage 25 embryo are shown with the location of interspersed repeats. (**c**) Mouse Hox gene clusters in the mm10 genome assembly. CTCF binding peaks in E14.5 embryonic tissue samples (brain and MEF) and ES cells are shown with the location of interspersed repeats. (**d**) Human Hox clusters in the hg19 genome assembly. CTCF peaks in GM12878 cells are shown with the location of interspersed repeats. (**e**) Dog Hox clusters in the CanFam3.1 genome assembly. CTCF peaks in the adult liver are shown. (**f**) Opossum Hox clusters in the MonDom5 genome assembly. Hox C genes were not identified as a cluster in the genome assembly. CTCF peaks in the adult liver are shown. For lamprey, chicken, and human, the summits of CTCF ChIP-seq peaks are shown differentially for two categories: "significant peaks" indicated with black bars and "consensus peaks" (only those with ≥5 fold of enrichments) indicated with gray bars (see Materials and Methods for the details of the definition of "significant peaks" and "consensus peaks"). For mouse and dog, peaks are shown for two categories, peaks with ≥10 fold of enrichments indicated with black bars, and peaks with ≥5 fold of enrichments with gray bars. For opossum, peaks with ≥5 fold of enrichments are indicated with black bars. Regions filled with undetermined bases are indicated in the row "N tract". In (**b**)-(**d**), all repeat categories are collapsed and shown in one row.

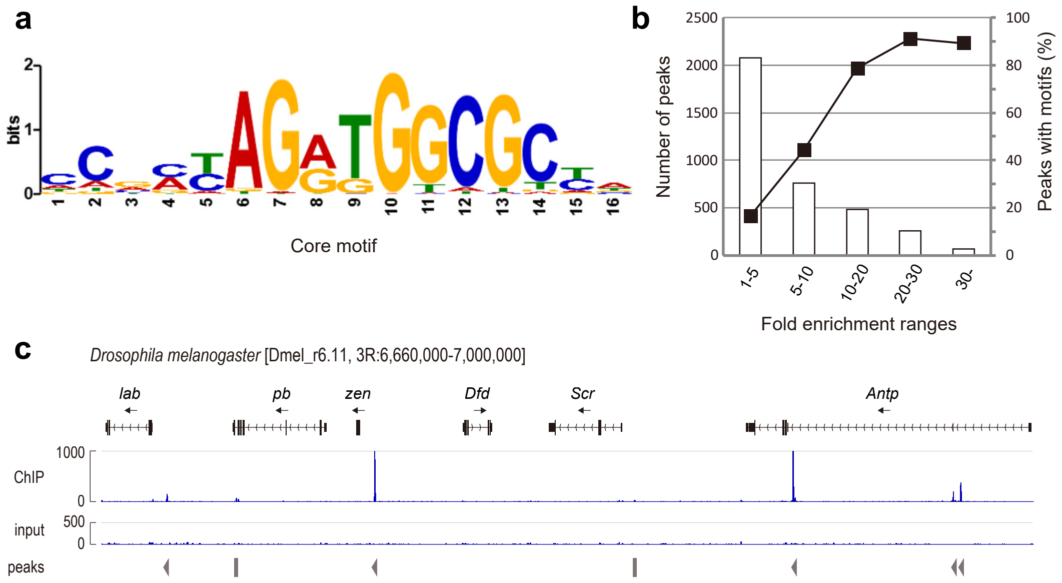

Fig. S8. Identification of CTCF ChIP-seq peaks and binding motifs in *Drosophila melanogaster*. (**a**) Top 500 peaks ranked in the order of fold enrichment were used for motif identification by MEME. (**b**) Numbers of ChIP-seq peaks at various fold-enrichment ranges are shown in white bars. Proportion of peaks containing the core motif is shown in a line. (**c**) ChIP-seq results at the *Antennapedia* complex (ANT-C). Peaks with more than 10 folds of enrichment were analyzed for core motif identification by FIMO. Gray arrowheads indicate peaks with the CTCF binding motif inside and its orientation, while gray bars represent peaks that contained no CTCF motif. Interestingly, all the identified CTCF motifs were oriented towards the 3′ end of the cluster. It was also shown that the ANT-C has two outstanding CTCF peaks, between *pb* and *Dfd* genes as well as within the *Antp* gene. The ChIP-seq data were obtained from NCBI SRA (SRR066831-SRR066836) and processed as described in Supplementary Materials and Methods. These data, originally in triplicates, were merged into one and mapped to the *D. melanogaster* genome assembly Dmel r6.11.

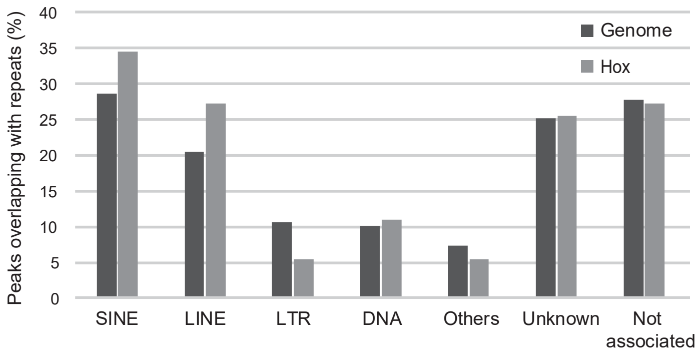

Fig. S9. Association of CTCF ChIP-seq peaks with interspersed repeat elements in Arctic lamprey Hox clusters. Significant peaks (peak summit ± 100 bp) in the whole genome of lamprey and in the Hox cluster region, were analyzed for their association with repeat elements. Number of peaks overlapping with repeat classes of SINE, LINE, LTR element, and DNA element were divided by the total number of peaks (27,633 in LetJap1.0, and 55 in Hox α~ε clusters). Overlap of ChIP-seq peaks to the repeats were 72.3 % (19981 out of 27633) genome-wide, and 72.7 % (40 out of 55) inside Hox α~ε clusters.

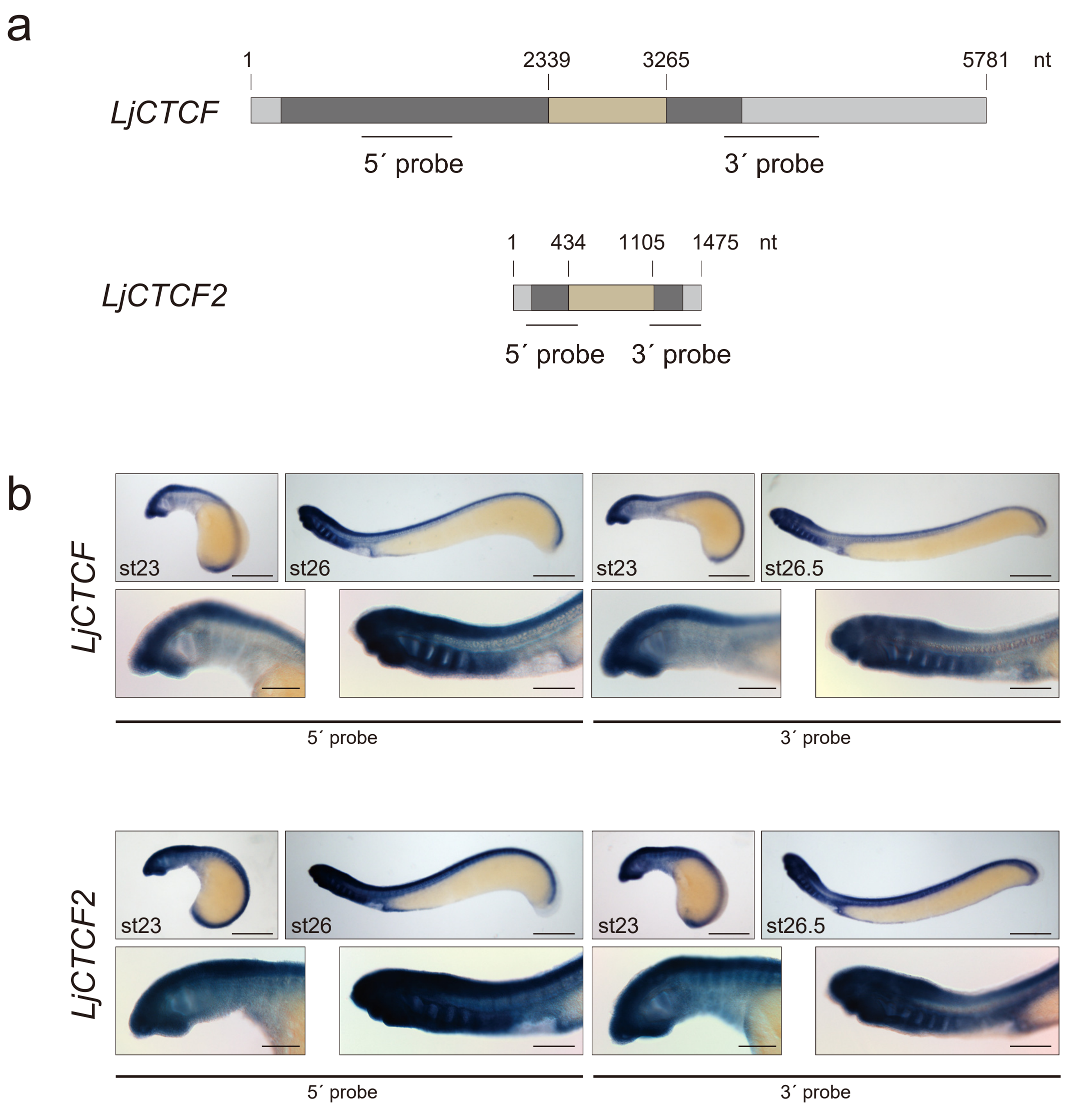

Fig. S10. Embryonic expression patterns of *L. camtschaticum CTCF* and *CTCF2*. (**a**) Locations of the two riboprobes (5´ and 3´ probes) designed for *CTCF* and *CTCF2* transcripts. Untranslated regions, Zn finger domains, and other coding regions are indicated in gray, brown, and dark gray, respectively. Templates of the riboprobes were amplified from the cDNA of stage 27 embryos with the oligonucleotide primers in Supplementary Table S7, and cloned into pCRII-TOPO vector (Thermo Fisher Scientific). Insert sequences were amplified by PCR with M13 reverse and forward primers, and 1.5 μg of the purified PCR product was used to generate Digoxigenin-labeled anti-sense cRNA probes with DIG RNA Labeling Kit (Roche Diagnostics). (**b**) Whole-mount *in situ* hybridization on *L. camtschaticum* embryos at stages (st) 23, 26 and 26.5. Photos are shown for whole bodies (top) and their magnifications (bottom). Results obtained for stage 26.5 embryos with the 3´ probe is included in Fig. 1c. Scale bars: 500 μm for the whole embryo panels, and 200 μm for the enlargement panels.

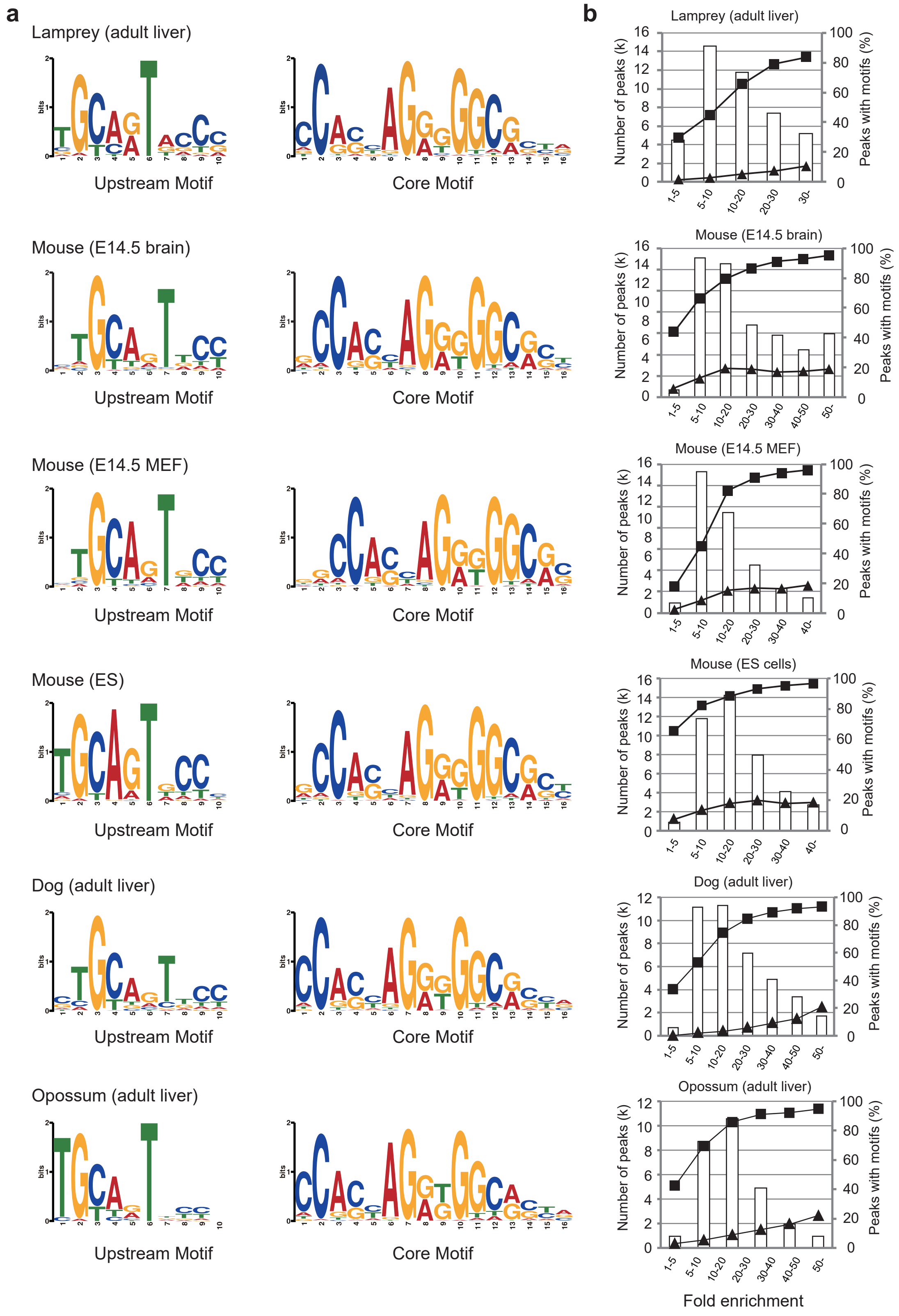

Fig. S11. Characterizing CTCF ChIP-seq peaks in adult lamprey liver, mouse E14.5 embryonic tissues, mouse ES cells, adult dog liver, and adult opossum liver. (**a**) Upstream and core CTCF binding motifs were identified for each of the samples from the top 2,000 ChIP-seq peaks ranked by fold enrichment. ChIP-seq data for mouse were obtained from NCBI SRA (SRR392354, SRR505014, SRR207080, SRR207071, SRR207089, and SRR207081). ChIP-seq data for dog and opossum were obtained from EBI ENA (ERR022285, ERR022304, ERR022303, ERR022307, ERR022306, and ERR022301). Data analysis for the lamprey liver was performed as described for embryonic samples in Materials and Methods. Data analysis for mouse, dog, and opossum were performed as described in Supplementary Materials and Methods. (**b**) Numbers of ChIP-seq peaks at various fold-enrichment ranges are shown with white bars. The proportion of peaks containing the core motif and core+upstream motifs are shown as lines with rectangles and triangles, respectively.

Table S1. RNA-seq read statistics.

Sample*	Number of sequence reads†		Number of mapped reads				
			Mapping to the whole genome‡			Mapping to coding sequences of predicted genes§	
	Raw reads	After adaptor and quality trimming	Mapped to LetJap1.0	Mapped to mtDNA	Total mapped reads¶	Uniquely mapped reads	Multi mapped reads
St.25 embryo	27,208,544	27,177,307	20,778,876	1,195,521	21,974,397	7,570,402	3,407,212
St.27 embryo	31,137,471	31,112,336	24,290,291	1,071,675	25,361,966	8,615,655	3,817,192
Liver	22,035,421	22,012,367	17,910,695	356,824	18,267,519	9,457,519	2,637,795
Eye	21,344,072	21,316,324	16,974,223	537,714	17,511,937	5,174,121	2,629,554
Brain	22,174,962	22,141,906	16,152,759	1,960,000	18,112,759	5,232,088	2,328,142
Intestine	23,666,023	23,647,364	16,696,683	1,127,148	17,823,831	5,503,856	3,431,200
Heart	21,762,562	21,747,218	12,047,351	4,107,753	16,155,104	4,528,816	2,167,198
Muscle	21,296,661	21,281,171	14,530,740	2,757,501	17,288,241	5,364,705	2,825,434
Testis	21,155,398	21,133,322	16,091,112	838,231	16,929,343	5,442,523	2,208,376
Oocyte	22,006,934	21,917,663	18,190,134	108,795	18,298,929	7,487,285	2,055,530

*Stage 25 and stage 27 RNAs were extracted from pooled embryos; testis RNA was from a single male; oocyte RNA from pooled unfertilized oocytes, and all other RNAs were from a single female.

†Single-end 80 nt reads were obtained in the Rapid Run mode of HiSeq1500.

‡Mapping was performed by Tophat2 without the use of gene model; unmapped reads were mapped to the mitochondrion genome (KF701113.1) by Bowtie.

§Mapping to coding sequences of the gene model based on AUGUSTUS (see Supplementary Materials and Methods) was performed by Bowtie2 with local alignment mode.

¶Sum of the numbers of reads mapped to LetJap1.0 and those mapped to mtDNA.

Table S2. Gene prediction statistics.

	Improved gene set	Original gene set
Number of genes	34435	34320
Completely reconstructed metazoan BUSCO genes	652	651
Missing metazoan BUSCO genes	94	95
Completely reconstructed CVG	209	208
Missing CVG	3	3
Curated prediction		
(i) split gene	254	
(ii) fusion gene	172	
(iii) truncated gene	6298	
(iv) newly predicted gene	110	

Table S3. Identifications of chicken and lamprey CTCF in immunoprecipitation.

a. Identified peptides in the Mascot search for chicken CTCF protein.

Position	Score	Peptide*
1-18	96	-.MEGEAVEAIVEESETFIK.G
2-18	73	M.EGEAVEAIVEESETFIK.G
23-27	12	R.KTYQR.R
145-168	29	K.GGLQEGEPMICHTLPLPEGFQVVK.V
169-193	35	K.VGANGEVETLEQGELQPQEDPNWQK.D
194-202	35	K.DPDYQPPAK.K
194203	44	K.DPDYQPPAKK.T
214-244	62	R.YTEEGKDVDVSVYDFEEEQQEGLLSEVNAEK.V
220-244	82	K.DVDVSVYDFEEEQQEGLLSEVNAEK.V
245-256	51	K.VVGNMKPPKPTK.I
264-277	119	K.KTFQCELCSYTCPR.R
265-277	65	K.TFQCELCSYTCPR.R
287-295	41	K.SHTDERPHK.C
311-323	57	R.NHLNTHTGTRPHK.C
324-324	95	K.CPDCDMAFVTSGELVR.H
345-352	30	K.HTHEKPFK.C
353-365	93	K.CSMCDYASVEVSK.L
372-389	53	R.SHTGERPFQCSLCSYASR.D
400-415	79	R.THSGEKPYECYICHAR.F
416-423	39	R.FTQSGTMK.M
424-429	37	K.MHILQK.H
430-436	37	K.HTENVAK.F
437-448	43	K.FHCPHCDTVIAR.K
449-457	55	R.KSDLGVHLR.K
450-457	50	K.SDLGVHLR.K
458-467	55	R.KQHSYIEQGK.K
459-467	33	K.QHSYIEQGK.K
471-479	59	R.YCDAVFHER.Y
480-487	38	R.YALIQHQK.S
495-505	60	R.FKCDQCDYACR.Q
497-505	58	K.CDQCDYACR.Q
509-514	35	R.HMVMHK.R
516-530	57	R.THTGEKPYACSHCDK.T
544-559	24	K.RYHDPNFVPAAFVCSK.C
545-559	56	R.YHDPNFVPAAFVCSK.C
563-566	17	K.TFTR.R
573-592	92	R.HADNCSGLDGGEGENGGETK.K
573-593	85	R.HADNCSGLDGGEGENGGETKK.G
654-665	12	R.GRPPGKAATQTK.Q
666-691	64	K.QSQPAAIIQVEDQNTGEIENIIVEVK.K

* Only the distinct peptide sequences are shown.

Mass spectrum was queried by NCBInr including the chicken CTCF sequence (NCBI NP_990663).

Mascot results: Score: 1348, Mass: 84315, Matches: 99(58), Sequences 40(31), emPAI: 3.4

b. Identified peptides in the Mascot search for lamprey CTCF protein.

Position	Score	Peptide*
23-46	29	R.LKEGGPPTQGTGLVDDDPTSNIPK.K
25-46	59	K.EGGPPTQGTGLVDDDPTSNIPK.K
47-70	38	K.KPEESEDGDLDQVPLVSEVALAVK.D
197-209	82	K.ESDDAFADAGSLR.V
289-312	68	R.DTGDETNSAEVDAVVESVDVNELK.A
507-514	45	R.GMLTEVVR.I
584-594	81	K.VGPNGELEVER.A
595-621	61	R.APMGSQDGSVDGDGIGLLITAPDPEER.S
624-634	50	K.EKDPDYQLPVK.K
669-679	63	R.LVSSQDVGVEK.A
680-690	27	K.AIAPKPPKPTR.I
698-711	35	K.KTFQCELCSYTCPR.R
699-711	61	K.TFQCELCSYTCPR.R
713-720	27	R.SNLDRHMK.S
736-744	14	R.AFRTVTLLR.N
739-744	14	R.TVTLLR.N
745-757	49	R.NHVNTHTGTKPHK.C
758-786	102	K.CMECDMAFVTSGELVR.H
779-786	31	R.HTHEKPFK.C
787-799	93	K.CSMCDYASVEVSK.L
806-823	41	R.SHTGERPFQCGLCSYASR.D
834-849	27	R.THSGEKPYECHVCHAR.F
850-857	41	R.FTQSGTMK.M
858-863	37	K.MHVLQK.H
864-870	39	K.HTDNVPK.Y
871-882	56	K.YHCPHCDAVIAR.K
883-891	60	R.KSDLGVHLR.K
884-891	59	K.SDLGVHLR.K
892-899	23	R.KQHAVLER.E
893-899	6	K.QHAVLER.E
908-913	12	R.AIFHER.Y
914-921	49	R.YALMQHQR.T
929-939	56	R.FKCDQCEYACK.Q
931-939	57	K.CDQCEYACK.Q
950-964	45	R.VHTGEKPFECTLCDK.T
979-993	60	R.YHDPSFVPTTYECSK.C
997-1000	17	R.NFTR.R
1007-1023	44	K.HFDMCDGELESGEQNGK.A
1091-1098	24	K.AKPGRPAK.K
1099-1107	36	K.KVAGSAEIK.T
1100-1107	21	K.VAGSAEIK.T
1100-1111	7	K.VAGSAEIKTEPK.T
1177-1193	105	K.EHVVVLAVETAADEGPK.N
1194-1207	69	K.NDITPEMILSMMDQ.

* Only the distinct peptide sequences are shown.

Mass spectrum was queried by LJ-GRAS including the lamprey CTCF sequences NCBI KX830966 and KX830967.

Mascot results: Score: 2082, Mass: 132346, Matches: 97(77), Sequences: 44(37), emPAI: 2.26

Table S4. ChIP-seq read statistics.

Sample	Source	Number of sequence reads*				Number of mapped reads†			
		Raw reads		After adaptor and quality trimming		Total mapped reads		Uniquely mapped reads	
		Input	ChIP	Input	ChIP	Input	ChIP	Input	ChIP
Lamprey_embryo_rep1	stage 27 embryos	16,272,896	15,843,949	16,183,265	15,451,120	10,823,154	11,221,293	9,962,493	10,521,159
Lamprey_embryo_rep2	stage 27 embryos	-	23,604,055	-	23,430,927	-	17,186,550	-	16,269,247
Lamprey_liver_rep1	adult liver	22,461,304	23,312,665	22,441,376	23,197,106	14,865,760	16,905,819	13,718,717	15,915,635
Lamprey_liver_rep2	adult liver	-	21,757,545	-	21,711,361	-	15,818,226	-	14,867,890
Chicken_embryo_rep1	stage 25 embryo	20,831,237	20,535,419	20,665,306	19,383,148	18,720,394	17,352,906	17,674,598	16,486,659
Chicken_embryo_rep2	stage 25 embryo	-	22,769,250	-	22,738,229	-	20,387,074	-	19,455,585
Human_GM12878_rep1‡	GM12878 lymphoblastoid cell line	23,479,237	22,031,342	23,000,738	21,893,794	21,543,427	20,854,117	19,688,311	19,336,104
Human_GM12878_rep2	GM12878 lymphoblastoid cell line	-	23,012,033	-	22,989,381	-	21,846,970	-	20,413,462

*Single-end 80 nt reads were sequenced by the Rapid Run mode of HiSeq 1500 except for human_GM12878_rep1.

†Lamprey and chicken ChIP-seq reads were mapped against LetJap1.0 and galGal5 genome assemblies, respectively, allowing multi-map (-m 5), whereas human reads were mapped against hg19 genome at single map (-m 1) condition of Bowtie (see Materials and Methods for details).

‡101 nt-long paired-end reads were produced by the High-Output mode of HiSeq1500, and only the read 1 trimmed to 80 bp were used for data analysis.

Table S5. CTCF peaks selected with various criteria.

Sample	Number of peaks called by MACS2		Number of selected peaks	
	Peaks in each replicate*	Peaks in merged replicates†	Consensus peaks‡	Significant peaks§
Lamprey_embryo_rep1	46,673	50,549	43,484	27,633
Lamprey_embryo_rep2	52,854			
Lamprey_liver_rep1	51,661	48,617	43,295	24,275
Lamprey_liver_rep2	50,872			
Chicken_embryo_rep1	36,412	48,269	33,554	16,382
Chicken_embryo_rep2	58,620			
Human_GM12878_rep1	58,856	59,754	53,066	29,542
Human_GM12878_rep2	63,551			

*Peaks were called for individual samples using the mapping result of input library for 'rep1' sample as control.

†Peaks were called for merged mapping results between both 'rep1' and 'rep2' samples using the mapping result of the input library for 'rep1' sample as control.

‡Peaks identified as intersect between peaks in 'rep1', peaks in 'rep2' and peaks in merged replicates.

§Subset of "consensus peaks" with their fold enrichment for "merged replicates" of ≥10 (for lamprey and chicken) and ≥20 (for human).

Table S6. Repeat contents of the whole genomes and Hox gene clusters of the four vertebrate species analyzed in this study.

Repeat categories*		*L. japonicum* (LetJap1.0)		*G. gallus* (galGal5)		*H. sapiens* (hg19)		*M. musculus* (mm10)	
Class	Sub-class	Genome	Hox α~ε †	Genome	Hox A-D †	Genome	Hox A-D †	Genome	Hox A-D †
SINE	Total	9.43	15.67	0.01	0	7.11	3.67	7.24	4.14
	ALU	0	0	0	0	5.96	3.43	4.87	3.04
	MIR	0	0	0	0	1.14	0.23	0.01	0.01
LINE	Total	12.05	8.59	7.05	0.04	20.36	5.81	18.58	1.28
	LINE1	0.01	0	0.06	0.02	19.36	5.69	18.08	1.06
	LINE2	2.16	1.46	0	0	0.99	0.11	0.46	0.21
	L3/CR1	0	0	6.92	0.03	0.01	0.01	0	0
LTR elements	Total	4.39	1.82	2.8	0.06	5.85	0.75	10.04	1.38
	ERVL	0	0	1.63	0.02	2.01	0.43	2.25	0.26
	ERVL-MaLRs	0	0	0	0	1.61	0.27	2.69	0.49
	ERV_class I	0	0	0.79	0.03	2.02	0.05	1	0.07
	ERV_class II	0	0	0.19	0	0.2	0	4.08	0.57
DNA elements	Total	4.06	3.71	1.02	0.16	2.24	0.64	1.12	0.32
	hAT-Charlie	0.53	0.32	0.15	0	0.8	0.18	0.33	0.04
	TcMar-Tigger	0.78	0.44	0	0	0.82	0.11	0.06	0
Unclassified		8.29	5.75	1.17	0.04	0.99	0.07	0.87	0
Total interspersed repeats		38.22	35.52	12.06	0.31	36.54	10.94	37.85	7.11
Small RNA		5.34	13.16	0.15	0.01	0.03	0.02	0.01	0
Satellites		0.9	0.64	4.82	0.07	0.07	0	0.33	0
Simple repeats		1.39	1.81	1.25	0.14	0.02	0	0.02	0.02
Low complexity		0	0	0	0	0	0	0	0
Total number of bases masked		40.35	37.6	18.18	0.53	36.5	10.93	38	7.06

RepeatMasker analysis was carried out for the whole genome and Hox clusters using the custom repeat library generated by RepeatModeler for each species. Shown are the percentages of repeat elements categorized into different (sub)classes.

*Repeat categories are based on the classification by RepeatModeler.

†Sequences between the 5′ and 3′ ends of the Hox clusters were analyzed.

Table S7. Oligonucleotide primers used.

Oligo name	Nucleotide sequence (5´-3´)	Application
Lj-CTCF-cDNA	GACACACTTTTCTCAAACCTCAG	cloning; gene specific RT primer (lamprey CTCF)
Lj-CTCF-ORF-f	GGCGAGGAGGAGCCGAGAATCT	cloning, PCR primer (lamprey CTCF)
Lj-CTCF-ORF-r	TGGAGTTTAGTTAGTTTACCCGCTGCGAT	cloning; PCR primer (lamprey CTCF)
Lj-CTCF-seq1	GATGGACGTTGTGGAAGTCA	Sanger sequence; sequence primer (lamprey CTCF)
Lj-CTCF-seq2	CTGGACGTGAGCACTAGCGT	Sanger sequence; sequence primer (lamprey CTCF)
Lj-CTCF-seq3	TGGATTGCTCATTACTGCCC	Sanger sequence; sequence primer (lamprey CTCF)
Lj-CTCF-seq4	CACGAGAAGCCCTTCAAGTG	Sanger sequence; sequence primer (lamprey CTCF)
Lj-CTCF-seq5	ACTTTGACATGTGCGATGGA	Sanger sequence; sequence primer (lamprey CTCF)
M13-forward	CGACGTTGTAAAACGACGGCCAGT	Sanger sequence; sequence primer
M13-reverse	GGAAACAGCTATGACCATG	Sanger sequence; sequence primer
AP	GGCCACGCGTCGACTAGTACTTTTTTTTTTTTTTTTT	3´ RACE; RT primer
AUAP	GGCCACGCGTCGACTAGTAC	3´ RACE; PCR primer
Lj-CTCF2-RACE-1	CCACCACGAGAAGCACTTCAA	3´ RACE; gene specific PCR primer (lamprey CTCF2)
Lj-CTCF2-RACE-2	GAAGAGCTCCATGAAATCCCA	3´ RACE; gene specific PCR primer (lamprey CTCF2)
Lj-CTCF2-cDNA	CTCACCTCACAAAGCCGTT	cloning; gene specific RT primer (lamprey CTCF2)
Lj-CTCF2-ORF-f	CCACCCGTCATTTATCGTTGCAGCAG	cloning, PCR primer (lamprey CTCF2)
Lj-CTCF2-ORF-r	CCTCACAAAGCCGTTCACTAACTCACCCAC	cloning; PCR primer (lamprey CTCF2)
Lj-Actb-f	GCTGGTTGTGTGGTGGAGG	qPCR validation; PCR primer (lamprey Actb)
Lj-Actb-r	TAACAAAAGACCGGCCGG	qPCR validation; PCR primer (lamprey Actb)
Lj-Hox-alpha7-f	GCCTCGGCTTATGCACCAG	qPCR validation; PCR primer (lamprey Hoxa7)
Lj-Hox-alpha7-r	CGCTGCCACGGCTCTTT	qPCR validation; PCR primer (lamprey Hoxa7)
Gg-Actb-f	CACCGAACGCTCCTCCCT	qPCR validation; PCR primer (chicken Actb1)
Gg-Actb-r	ACATCAGGTACGGCAACGG	qPCR validation; PCR primer (chicken Actb1)
Gg-HoxB8-f	CAACACCTTGATTTCATTAGCGG	qPCR validation; PCR primer (chicken HoxB8)
Gg-HoxB8-r	TCGGGATGGTTCCTGCTC	qPCR validation; PCR primer (chicken HoxB8)
Hs-Gapdh-f	CCACATCGCTCAGACACCAT	qPCR validation; PCR primer (human Gapdh)
Hs-Gapdh-r	AGCCACCCGCGAACTCA	qPCR validation; PCR primer (human Gapdh)
Hs-H19-1f	GCAGAAYCGGTTGTAGTTGTGG	qPCR validation; PCR primer (human H19)
Hs-H19-1r	TGATGTGGGAGCCTGCAC	qPCR validation; PCR primer (human H19)
Hs-H19-2f	CCCGTGGAAACATCCCAG	qPCR validation; PCR primer (human H19)
Hs-H19-2r	TATACCTCACGACCCCTGTGAAC	qPCR validation; PCR primer (human H19)
Hs-HoxA7-f	CATATAGGCTCTGGGTGATCGC	qPCR validation; PCR primer (human HoxA7)
Hs-HoxA7-r	ATTTCCCAGCAAGCAGGGTA	qPCR validation; PCR primer (human HoxA7)
#274-DNA-f	CCAGCCACTAAGCGACCCTT	qPCR validation; PCR primer (lamprey hAT-Tip100)
#274-DNA-r	ACTCTAGGGGGCCCTCTGCT	qPCR validation; PCR primer (lamprey hAT-Tip100)
#274-DNA-1f	GAGCACCGCCGCCTATT	qPCR validation; PCR primer (lamprey hAT-Tip100)
#274-DNA-1r	AACCAAATTTTGCTTAGGGCC	qPCR validation; PCR primer (lamprey hAT-Tip100)
#274-DNA-2f	GCCGAGTTTATTCAATCCCG	qPCR validation; PCR primer (lamprey hAT-Tip100)
#274-DNA-2r	CAAATTTTGCTTAGGGCCCC	qPCR validation; PCR primer (lamprey hAT-Tip100)
#274-DNA-3f	GCAATTGCAAGGGTCGCTTA	qPCR validation; PCR primer (lamprey hAT-Tip100)
#274-DNA-3r	TCTCCCTCGTATTGCCATGAC	qPCR validation; PCR primer (lamprey hAT-Tip100)
#274-DNA-4f	CGCATGGCATCCTTGTTG	qPCR validation; PCR primer (lamprey hAT-Tip100)
#274-DNA-4r	TGCAAGGGTCGCTTAGTGG	qPCR validation; PCR primer (lamprey hAT-Tip100)
#274-DNA-5f	GCAATTGCAAGGGTCGCTTA	qPCR validation; PCR primer (lamprey hAT-Tip100)
#274-DNA-5r	GACAGGATTCTGGAAGGCCA	qPCR validation; PCR primer (lamprey hAT-Tip100)
#274-DNA-6f	GCAATTGCAAGGGTCGCTTA	qPCR validation; PCR primer (lamprey hAT-Tip100)
#274-DNA-6r	CTGTAGATGCAGAACAGCGCA	qPCR validation; PCR primer (lamprey hAT-Tip100)
#274-DNA-7f	GCAATTGCAAGGGTCGCTTA	qPCR validation; PCR primer (lamprey hAT-Tip100)
#274-DNA-7r	CGTCGTTTATGCGTTTCTCTCA	qPCR validation; PCR primer (lamprey hAT-Tip100)
#274-DNA-8f	TGTGACCACGACACAAAAGCA	qPCR validation; PCR primer (lamprey hAT-Tip100)
#274-DNA-8r	AGCGGTCATACACGCCAGTAA	qPCR validation; PCR primer (lamprey hAT-Tip100)
#274-DNA-9f	ATGGAATCGTGCCCGTGAT	qPCR validation; PCR primer (lamprey hAT-Tip100)
#274-DNA-9r	CAATTGCAAGGGTCGCTTAGT	qPCR validation; PCR primer (lamprey hAT-Tip100)
#274-DNA-10f	TCGTGGGCTTCGGTTTTG	qPCR validation; PCR primer (lamprey hAT-Tip100)
#274-DNA-10r	GCCGGCTCTGGGAAGAA	qPCR validation; PCR primer (lamprey hAT-Tip100)
#274-DNA-11f	GCAATTGCAAGGGTCGCTTA	qPCR validation; PCR primer (lamprey hAT-Tip100)
#274-DNA-11r	ACTGTATATGCTTCTGTGTCTCTGTGTAGT	qPCR validation; PCR primer (lamprey hAT-Tip100)
#274-DNA-12f	CCATCACGTACAACTGCCGA	qPCR validation; PCR primer (lamprey hAT-Tip100)
#274-DNA-12r	AAGGTCTAGGGCCGGTTCTG	qPCR validation; PCR primer (lamprey hAT-Tip100)
CTCF_ISH_5prime-f	AGCGTGGAGACCATGGATAC	ISH; PCR primer (CTCF 5´ probe)
CTCF_ISH_5prime-r	CTCCTCCTTCTCCCGTCTCT	ISH; PCR primer (CTCF 5´ probe)
CTCF2_ISH_5prime-f	AGGTCCCAGCAAGAGCAAT	ISH; PCR primer (CTCF2 5´ probe)
CTCF2_ISH_5prime-r	GTACCGGTGTGGGTGTTGAT	ISH; PCR primer (CTCF2 5´ probe)
CTCF_ISH_3prime-f	GGGGGAAGAACAGGAAGAAG	ISH; PCR primer (CTCF 3´ probe)
CTCF_ISH_3prime-r	AATGTAAATCGGCGATGCTC	ISH; PCR primer (CTCF 3´ probe)
CTCF2_ISH_3prime-f	GCAGGAGCGACACATGATAG	ISH; PCR primer (CTCF2 3´ probe)
CTCF2_ISH_3prime-r	CGCTCATTAAACACCCACTG	ISH; PCR primer (CTCF2 3´ probe)

Table S8. Sequence IDs used for molecular phylogenetic analysis.

Classification	Gene name	Species		Accession ID		Notes
		Common name	Scientific name	Nucleotide	Amino acid	
CTCF	CTCF	human	*Homo sapiens*	NM_006565.3	NP_006556.1	
	CTCF	ostrich	*Struthio camelus*	Sca_R014733		identified in Struthio_camelus.OM.gene.20130116.pep transcript assembly*
		Chinese soft shell turtle	*Pelodiscus sinensis*	ENSPSIT00000003816	ENSPSIP00000003796	
	CTCF	green sea turtle	*Chelonia mydas*	XM_007067168.1	EMP28837.1	
	ctcf	western clawed frog	*Xenopus tropicalis*	ENSXETT00000034066	ENSXETP00000034066	
		coelacanth	*Latimeria chalumnae*	ENSLACT00000011258	ENSLACP00000011174	
		three spined stickleback	*Gasterosteus aculeatus*	ENSGACT00000003281	ENSGACP00000003270	
		three spined stickleback	*Gasterosteus aculeatus*	ENSGACT00000020979	ENSGACP00000020939	
		elephant shark	*Callorhinchus milii*	SINCAMT00000007220	SINCAMP00000007143	identified in eshark_proteins.fa transcript assembly †
	CTCF	small-spotted catshark	*Scyliorhinus canicula*	comp71432_c0_seq1		identified in Sca_all_tissues300.fas transcript assembly ‡
	CTCF	inshore hagfish	*Eptatretus burgeri*			see Supplementary Table S9a for nucleotide and amino acid sequences
	CTCF	Arctic lamprey	*Lethenteron camtschaticum*	KX830966		sequence determined in this study
	CTCF	sea lamprey	*Petromyzon marinus*	ENSPMAT00000004708	ENSPMAP00000004689	
	zf(c2h2)-30	vase tunicate	*Ciona intestinalis*	NM_001111123.1	NP_001104593	
			Oikopleura dioica		CBY12384	
		lancelet	*Branchiostoma floridae*	e_gw.69.11.1		see Supplementary Table S9b for nucleotide and amino acid sequences; idenfiied in Brafl1.FilteredModels1.gff § and modified incorporating GENESCAN prediction
CTCFL	CTCFL	human	*Homo sapiens*	NM_080618.3	NP_542185.2	
	CTCFL	ostrich	*Struthio camelus*	Sca_R006826		see Supplementary Table S9c for nucleotide and amino acid sequences; identified in Struthio_camelus.OM.gene.20130116.pep transcript assembly* and modified incorporating GENESCAN and AUGUSTUS prediction
		Chinese soft shell turtle	*Pelodiscus sinensis*	ENSPSIT00000012554	ENSPSIP00000012493	
		green sea turtle	*Chelonia mydas*	XM_007059856.1	XP_007059918.1	
CTCF2	CTCF2	Arctic lamprey	*Lethenteron camtschaticum*	KX830967		sequence determined in this study
	CTCF2	sea lamprey	*Petromyzon marinus*			see Supplementary Table S9d for nucleotide and amino acid sequences

* http://gigadb.org/dataset/101013

† http://esharkgenome.imcb.a-star.edu.sg/

‡ http://datadryad.org/resource/doi:10.5061/dryad.s1f22

§ http://genome.jgi-psf.org/Brafl1/Brafl1.home.html

Table S9. Nucleotide and deduced amino acid sequences identified in this study.

a

Species	Gene	Type	Sequence
Eptatretus burgeri (inshore hagfish)	*CTCF*	Nucleotide coding sequence	ATGTCTAAATCCAGCCCTGTGCGGAGAGCGGACCCAAATGCAATACCCTCTTCTCCGGTAATGGCATCAGATGAA<br>GAAGAAACGGTGTTACCATCTTCAAAGTCTTCAAACATCTTCACAAAAGTGAAGGATGCAGAAAGTGCCCAGTTT<br>GAAGTGGTCATGACTCTTGACGACGTACCCAAACAGAAGATAGAAAATGAGGAAACGGATGTACAGGATATGAGA<br>ACAACACACTTCATTTGCGGTTTGCAGATGCCTGAGCAGCAGCAAGGAGAATGTGAAACTATTGATGACACTGCT<br>AATGCTGTTAATGAAGGCACTAGTGTCCAAGAGGGCATGGCACTTACCGTTCAGGCGCTGCAGGCCCTCCAGCAC<br>GTGACTGTTGATCAAGGCACCAGCGATGATACGCACATAATTACACTCCATCCCGTAAGCCTGGAGGAGGCAACA<br>TCTGGGGGTGCTTCCTCTCTCAGTGCCATCACCCTTGTGAGGTTGCCTACCGAAGATACTGGCACCACCATGCAC<br>CGCTTATCCAGCGATCCTGGGATTATGGCGACAGCTGTAACCACTTCAGTAGTATCGGGATCAACCACTGAAGGC<br>CTGGTGGTGCATGTTGGCTCTGGAGGCCTTGTTGCTGAGGTCGTAGGCGTATCAGAGGATGGAACTGTAACAGTC<br>GGAACGGGCGACGATGGAGGCAGTAGCATTGTGACTGAAGGTCTTGTCACTGAGATCATTCCTATCAACCAGGCT<br>GGTACCTCTGAGCAGGGGCAAGGACCCATCATCAGCCACACACTCCCACTTCCCATGGGGGTTCAGGTGGTAAAG<br>GTGGGGCCCAATGGAGAGGTGAGCATGGAAGATGATGCCGAATTGGAAAACATGGTCGATGGTACACCAATGGAA<br>AGTCTTGAGGATTCTAAAGTCCATCTCGCTGGAAGCCTGGAAGTTACTGTGCATGATGGGGTGAACATGGCAGTT<br>GAGATCAAGCCTTCTGTGGATGAAGAAGAATCGACGAAGGAAGAGAGTGAGGAGAAGGAAAAGCAAAGTGATCCT<br>GATTATCAGCTTCCAAGCAAGAAGCCTATCCGCAAGGGCAAGAGGAACAAGCTGCGGTACAAGGTTGAAGAAGCA<br>AATGAGCAAGACATATCCGTGTACGATTTTGAGGACCAGGAGGGTGAACGTCTCGTGAGCAGTCAGGACGTCGGT<br>GTGGAGAAGGCCATTACGCCCAAACCGCCAAAGCCAACTAAGATCAAAAAGAAAGGTGCCAAGAAGACGTTCCAG<br>TGTGAGCTCTGCAGCTATACATGTCCACGGAGGTCGAACCTTGATCGACACATGAAGAGCCACACGGATGAGCGG<br>CCTCATCGTTGCCACCTTTGTGACCGTGCTTTTCGCACCGTCACGCTACTCCGCAACCATGTGAACACGCATACA<br>GGCACAAAGCCACACAAATGCCCCGAGTGCGACATGGCTTTTGTGACAAGTGGAGAGTTGGTTCGGCACAGGAGA<br>TACCGCCACACGCACGAGAAACCCTTCAAGTGCTCCATGTGTGATTATGCCAGTGTGGAGGTCAGCAAGTTAAAA<br>AGGCATATCCGATCTCACACCGGAGAGCGTCCATTTCAGTGTGGCCTTTGCAGCTATGCTAGCCGTGATACGTAC<br>AAGCTGAAACGGCATATGCGTACACACTCGGGGGAAAAGCCTTATGAATGCCATATATGCAATGCACGCTTCACC<br>CAGAGCGGCACCATGAAGATGCATGTGCTGCAAAAGCACACTGAGAATGTGCCAAAGTACCACTGCCCCCACTGC<br>GATGCCGTCATTGCCCGCAAGAGTGACCTTGGTGTCCACCTGCGTAAGCAACACTCAATTGTGGAGCGTGAATTG<br>AAGTGCCGATACTGTGACCTGATATTCCATGAACGCTATGCCCTCATGCAGCACCAGCGCTCACACAAAAATGAG<br>AAGCGATTCAAATGTGATTGCTGTGAATACTCCTGCAAGCAGGAGAGGCACATGGTAATGCACAAACGTGTGCAC<br>ACTGGAGAGAAGCCCTTTGCCTGCATGCTTTGTGACAAGACCTTTCGACAGAAGCAGCTGCTTGACTTCCACTTC<br>AAGCGTTACCATGACCCTAACTTCATCCCCACCACGTACGAGTGTGAGAAATGTCACAAGGCCTTCACCCGCAGG<br>AACACTATGATGAAGCACAGCGAAAACTGCGATGGTGAAGTAGACGGAGAGCAGAATGGGCGTGGTCGACGACGT<br>CACCGATGTGGCCGCAAGCGGAAGATGCAAAGGCGCAAGCAGGATTTATCCGATTCGGATGTGGATCCTGTAAGT<br>GAGGATGAGGTTGAAGAGGAGGGTGAAGTGGAGGAAGAAGAAGAGGAAGAGGCTGAAGCTGTTCCACCGCCTGTC<br>AAACGCCGCCGTGGTCGACCTCCCAAGGCCAGACCATTTGTTGAAGTACAACATAAAACGGAAGTGAAAGAGGAG<br>CCGATCGATTTGGATTCAACCATCAACACAGTGGCAAGTGGAATAGTGGAGATAATTCCTGTGACGGTGCAAGGA<br>GGGTCAGACAGTGATCAAGGCGGTGAAGGGACCGTTGCAAAGCCTGAGGAAGAAGTTGAGGGAACAGAACAGGTG<br>GCCACTATTGTGATAGAAGCAGCAGGGAACAGTGGTGAACGGTCAGACATCACCCCAGAGATGATTCTAAGCATG<br>ATGGACCAGTGA
		Deduced amino acid sequence	MSKSSPVRRADPNAIPSSPVMASDEEETVLPSSKSSNIFTKVKDAESAQFEVVMTLDDVPKQKIENEETDVQDMR<br>TTHFICGLQMPEQQQGECETIDDTANAVNEGTSVQEGMALTVQALQALQHVTVDQGTSDDTHIITLHPVSLEEAT<br>SGGASSLSAITLVRLPTEDTGTTMHRLSSDPGIMATAVTTSVVSGSTTEGLVVHVGSGGLVAEVVGVSEDGTVTV<br>GTGDDGGSSIVTEGLVTEIIPINQAGTSEQGQGPIISHTLPLPMGVQVVKVGPNGEVSMEDDAELENMVDGTPME<br>SLEDSKVHLAGSLEVTVHDGVNMAVEIKPSVDEEESTKEESEEKEKQSDPDYQLPSKKPIRKGKRNKLRYKVEEA<br>NEQDISVYDFEDQEGERLVSSQDVGVEKAITPKPPKPTKIKKKGAKKTFQCELCSYTCPRRSNLDRHMKSHTDER<br>PHRCHLCDRAFRTVTLLRNHVNTHTGTKPHKCPECDMAFVTSGELVRHRRYRHTHEKPFKCSMCDYASVEVSKLK<br>RHIRSHTGERPFQCGLCSYASRDTYKLKRHMRTHSGEKPYECHICNARFTQSGTMKMHVLQKHTENVPKYHCPHC<br>DAVIARKSDLGVHLRKQHSIVERELKCRYCDLIFHERYALMQHQRSHKNEKRFKCDCCEYSCKQERHMVMHKRVH<br>TGEKPFACMLCDKTFRQKQLLDFHFKRYHDPNFIPTTYECEKCHKAFTRRNTMMKHSENCDGEVDGEQNGRGRRR<br>HRCGRKRKMQRRKQDLSDSDVDPVSEDEVEEEGEVEEEEEEEAEAVPPPVKRRRGRPPKARPFVEVQHKTEVKEE<br>PIDLDSTINTVASGIVEIIPVTVQGGSDSDQGGEGTVAKPEEEVEGTEQVATIVIEAAGNSGERSDITPEMILSM<br>MDQ

b

Species	Gene	Type	Sequence
Branchiostoma floridae (lancelet)	*CTCF*	Nucleotide coding sequence	ATGGCGACACCTAGGCTACTGTGGAACATCCGTGCGCCTGTCCAATTGATTGAAAGAGATGGTAGAAAAGGTGGC<br>GTCTTCCTTGTCTCATCGGGGGTCATCCGAAAAGATCCTGGTAGTTTCACAAAACGGAAGAGACAACGACAAAAC<br>AAAAGTCCAATAGCACTGCCAATAACTACATCTGGCCACCCCTTCCTCACCGCGTCTCTCTCCGCATTCGTGGCA<br>AATATCGCCGAACATTCGCCATTTTGGGCGGCAATCTTCGCCGTTTCTGCCCGCCTCTGCCTCGGAACCCTTCCA<br>AACACCACGCTTCTGACCGGGGGATTCGTTCTCCGGAGCCTGGACGGCACTTTTGCGACTTGTTTTGGAGGAGAG<br>GAGAAAAACCAAATTAAGAAGAAAGTAGCTCGGTCACTCGCGGAGAGGGAGCTTGTACCACGTGTCAAATATAAG<br>CCACTACTACAAAGTCCGCTAGTGCGGAGTCCCGTCTCTTATACGAACATTGCTTTACATGCGCATCCTGTCCAG<br>GAAGAAACTGAAGATAGTTCAGCTGAACAGGTGCAGGAGGCAGTTGAACAGGTGGAAGCACCTGGTGACCAGGTG<br>GAGGCAGCAGCTGAACAGGTGCAAGCACCAGGTGAACAGGGGGAGGCATCAGGTGAACAGGTGCCAGCCGAGAAG<br>CAGGACGACCTGGGACAGCTGCAGTCGTACCTGGAGACATTCCACCAGGAGATCTCGGCAGATGTCAAGCCCCTG<br>GCTGAGGGGGGTGCTGACGGACAGCAGGGTAAAGATGAGGCACCAGCAGATGCTGTTCCTGCTGTGGAAGAAGCA<br>GCTGCTCCTGTTGAGAGCACAGGTGAAGAAACAGCTGCACCTGAAGAAATGGAAACAACCCAAGGTGTCGAGGAG<br>GCAGAGAAGTCTCCAGCCGAGGCACCTGCAGAGGATGAAGCACCTGATGCGGCTGGAACTTCAGCTGTAGAAGAA<br>GCAGCACCTGCTGAGCAGGTGTATGTATCAGCTGAAACCACAGGGGAAGCAGCTGCACAGGAGACCCAGGGACAA<br>CAACAACCACAGCAGGTTCGCATTGTCACCACCCAGGGGGGGACAGAAGTTCATGAGGTCGTCAGCGAGGAGGTT<br>GCAGCACAGCTTCAGCAGATGGCCGCGGAGAGTGGCGGTACGGGATCGGTCAGCATCATCGTGCAGAGCGGCTCA<br>GACGAGGGGCAGGTCACGGCGAGCCAGGCGGTGGGCTCGCTGCAGCCCGGACAGATCACGGTGATGTACGAGCTG<br>GTTCCCGTGGACATCGCGCAGGGGGAGGGGCAGCAGCAGGCGGCACAGCCTGTGGAGAATGGGGAGGAGGAAGAG<br>CCGGAAGCTGAGGACCAGGAGGGGGAGGAGCCAGATGGAAGTGCAGCCGACCCTGACTGGCAGGAGGGGGAGGAT<br>GGGGGGGCGGAGTCCACCAAGGAAGGGGACATCTCGGTCTACGACTTCGATGAGCTAGACAAACCGGATGGAAA<br>CGCTCAAAGGGAGGAGACGCGATGACCCCCAAGGTCAAGGGGAGGTCACCAAAGAAGATGCGTGTTGGTGGGAAG<br>ACGTACCAGTGCTACAAGTGCGACTACACGTGTCAGAGGATGGCCTTCCTGGAGCGCCACATGAAGGTGCACACG<br>GACGAGCGCCCCTTCAAGTGTGGCACGTGCGAGCGGGAGTTCCGCACCATGCAGTCCCTCCAGAACCACATCAAC<br>AGCCACAACGGGGTCAAGCCCCACAAGTGCGACCAGTGCCCCATGAGCTTTGTGACGAGCGGGGAGCTGATGCGC<br>CACCGACGGTACAAGCACACGCACGAGAAGCCGCACAAGTGCACCATGTGCGACTATGCCAGCGTGGAGATCAGC<br>AAGCTCAAGCGCCACATGAGGTCTCACACCGGAGAGCGACCCTTCCAATGTGGCATGTGTAGCTATGCGAGTCCT<br>GACAGCTACAAGCTGAAGCGCCACATGCGCACCCATACAGGCGAGAAGCCATACGAGTGCTCCGTGTGTCTCGCC<br>ACCTTTACCCAGAGTGGCAGCCTCAAAATGCACATGCAGCGTCATTTGGGAACGGCGCCCTCTTACGTCTGCGAC<br>ATCTGTGGCACGGCGCTGACGCGGAAGAGCGACCTGAAGTCTCATGTCCGCAAACTCCACACCGGCGACAAGCTG<br>CTCACCTGCAAGTACTGTGACTCAGCCTTCCCCGACAAGTACAACCTTACCAAACATCTCAAAACACACCAGGTC<br>AGTACAACCTTACCAAACATCTCAAAACACACCAGTTCAGTACAACCTTACCAAACATCTCAAAACACACCAGTT<br>CAGTACAACCTTACCAAACATCTCAAAACTCACCAGGTCAGTACAACCTTACCAAATATCTCAGTACAACCTTAC<br>CAAACATCTCAAAACACACCAGGTCAGTACAACCTTACCAAACATCTCAAAACACACCAGGTCAGTACAACCTTA<br>CCAAACATCTCAAAACACACCAGGTCAGTACAACCTTACCAAACATCTCAAAACACACCAGGTCAGTACAACCTT<br>ACCAAACATCTCAAAACACACCAGGTCAGTACAACCTTACCAAACATCTCAAAACACACCAGGTCAGTACAACCT<br>TACCAAACATCTCAAAACACACCAGGTCAGTACAACCTTACCAAACATCTCAGTACAACCTCAACAAACATATTA<br>CAACACACCTGGTTTGTACAACCTTATCGAACATCTCAGTACAACCTCAGCAAACATATTACAACACACCTGGTT<br>TGTACAACCTTATCGAACATCTCACAGTACAACCTCAGCAAACATATTACAACACACCTGGGAGAGAAGCGCTTC<br>AGGTGTGAGGACTGTAACTACTGCTGTACCCAGGAGAGGCACCTGATCAACCACAAGCGCTGCCACACCGGCGAG<br>AAACCCTTCGTCTGTGTGCAGTGTGACCACACCTTCCGCCAGGAGCAGCTGCTCAAGCAGCACATAAAGGTGCAC<br>CACACGCCTGGCTACACCCCTCCCCGCTACGCCTGCACCAACTGCGACAAGTCCTTCACCCGCAAGGGGAACCTG<br>CGCAAGCACGTGGAGCAGGCGCACGACCCCAGCGCCGTGCTGCCCGCCATGATGGGGCGCGGCAGCAGGGGGTCG<br>CGGCTACACGACCGGCTGGACGATGATGATTCCATGTCGGAGGGGGAACACGAGTTTGAGCAGGCCATGGACGAG<br>ATCGAGGCACAGCAGATGGACGGAGAGGGCATGGAGGAGCGGGAAATGGAGCCGCAGGATGAAGACTCAGATGCC<br>GACTACAGGCCAGGAGACGACATCGACGAAACGCCCAAGCGCAAGCCCAAGACCCCCCGTAAGAGGAAAGCCAAC<br>ACGCCCAAGTCCACGCCCCAGAAGAGGAAACGACAGAAGAAGAAGATTAAGTCTGAGGAGGAGGAGGCTGAGGAG<br>GAACCGGAGGGGGAGGAGATGGGAGAGGCTCCTCCTCCTGCCACTAAGGAAGGAGAACAGCCAGCAGAGGAAGCT<br>GAAGCTGAAGCAGCTGTGGAGGATCAGCAACCAGCTGAGGCTGAGGCTGAAGCTGAGGCTGAAACTGAAGCTACA<br>GAGCAGACCTAG
		Deduced amino acid sequence	MATPRLLWNIRAPVQLIERDGRKGGVFLVSSGVIRKDPGSFTKRKRQRQNKSPIALPITTSGHPFLTASLSAFVA<br>NIAEHSPFWAAIFAVSARLCLGTLPNTTLLTGGFVLRSLDGTFATCFGGEEKNQIKKKVARSLAERELVPRVKYK<br>PLLQSPLVRSPVSYTNIALHAHPVQEETEDSSAEQVQEAVEQVEAPGDQVEAAAEQVQAPGEQGEASGEQVPAEK<br>QDDLGQLQSYLETFHQEISADVKPLAEGGADGQQGKDEAPADAVPAVEEAAAPVESTGEETAAPEEMETTQGVEE<br>AEKSPAEAPAEDEAPDAAGTSAVEEAAPAEQVYVSAETTGEAAAQETQGQQQPQQVRIVTTQGGTEVHEVVSEEV<br>AAQLQQMAAESGGTGSVSIIVQSGSDEGQVTASQAVGSLQPGQITVMYELVPVDIAQGEGQQQAAQPVENGEEEE<br>PEAEDQEGEEPDGSAADPDWQEGEDGGAESTKEGDISVYDFDELDKPDGKRSKGGDAMTPKVKGRSPKKMRVGGK<br>TYQCYKCDYTCQRMAFLERHMKVHTDERPFKCGTCEREFRTMQSLQNHINSHNGVKPHKCDQCPMSFVTSGELMR<br>HRRYKHTHEKPHKCTMCDYASVEISKLKRHMRSHTGERPFQCGMCSYASPDSYKLKRHMRTHTGEKPYECSVCLA<br>TFTQSGSLKMHMQRHLGTAPSYVCDICGTALTRKSDLKSHVRKLHTGDKLLTCKYCDSAFPDKYNLTKHLKTHQV<br>STTLPNISKHTSSVQPYQTSQNTPVQYNLTKHLKTHQVSTTLPNISVQPYQTSQNTPGQYNLTKHLKTHQVSTTL<br>PNISKHTRSVQPYQTSQNTPGQYNLTKHLKTHQVSTTLPNISKHTRSVQPYQTSQNTPGQYNLTKHLSTTSTNIL<br>QHTWFVQPYRTSQYNLSKHITTHLVCTTLSNISQYNLSKHITTHLGEKRFRCEDCNYCCTQERHLINHKRCHTGE<br>KPFVCVQCDHTFRQEQLLKQHIKVHHTPGYTPPRYACTNCDKSFTRKGNLRKHVEQAHDPSAVLPAMMGRGSRGS<br>RLHDRLDDDDSMSEGEHEFEQAMDEIEAQQMDGEGMEEREMEPQDEDSDADYRPGDDIDETPKRKPKTPRKRKAN<br>TPKSTPQKRKRQKKKIKSEEEEAEEEPEGEEMGEAPPPATKEGEQPAEEAEAEAAVEDQQPAEAEAEAEAETEAT<br>EQT

c

Species	Gene	Type	Sequence
Struthio camelus (ostrich)	*CTCFL*	Nucleotide coding sequence	ATGCAGAATGTTGACTTGGCAGAAAGAAATGGACCCTTCAGAGGAGTAGGCGTGGTCCTGAGCGTACAGGAAGGT<br>GTCTGCGCGTTTTACGACTTGGAGTTGATGCAGATTAGTATTTTGCAAGAAAAAGCGCAAGCGAAGAATTTAGAA<br>AGCAAGTCAGCAGAGAGACCGCTAGACATGCTACTGATTGAGCTTGACAGAGATAAAGATGTCATTGCACTTGAA<br>AATAAGGTCCAGCCACTAACCCCATCTGAAGAAGGAGGAGAAAAGGAACTTTTCAGTGTTAGGGAAGCAAAGAGC<br>CGGGACTGTAAAGCCAGTGGTGATTTGTTGGTTTCATGCAATAAATATAAAGAACAGGAATACGTGACTCCGTCA<br>GAGCAAGCTGTAACAAAAAATCCCAAGTCTCAAAAATCTCAGAAAGGTAAAAAAGCAGTTTTCAGCTGTGATTTG<br>TGCACATTCACCTCACTCAGAATATCAAGTCTTAACCGTCACATGAAAACCCATTCGGATGAAAAACCTCATGTG<br>TGTCACCTCTGCCTTAAGGCTTTTCGTACAGCTACTCTCCTGCGAAACCATGTGAATGCACATACAGGGACCAGA<br>CCGTATAAATGCAGTGACTGTGATATGGCATTTGTGACCAGTGGTGAGCTTGCGCGACACAGACGTTACAAGCAT<br>ACTTTAGAAAAACCCTTCAAGTGTTCAATATGTAAATATTCTAGCGTAGAAGCAAGCAAACTGAAGCGACACATA<br>CGCTCGCATACAGGAGAGCGTCCTTACGCCTGTTACCTTTGCAGTTATGCTAGCAAAGATACATATAAACTGAAA<br>AGACACATGATAACTCATTCAGGTGAAAAACCATATGAATGTTATGTTTGCCAGGCCAGATTCACTCAAAGTGGT<br>ACCATGAAAATCCATATATTGCAAAAGCATGGTGAAAATGTGCCAAAATACCAGTGTCCACATTGTAATACATTT<br>ATTGCACGAAAAAGTGACTTGGGTGTCCACTTGCGAAATCTGCATTCCTACATGGCAGTGGCAATTAAATGCAGT<br>TACTGTGAAGCTGTTTTTCATGAGCGCTATGCTCTTACTCAGCACAAGAAGACTCACAAAAATGAAAAAAGATTC<br>AGATGTGATCAGTGCAGCTATGCATGCAAGCAGGAGCGACACTTAATTGTACATAAACGAACCCATACTGGTGAG<br>AAGCCCTTCACTTGCTTGTGCTGCAGCAAAAGCTTTCAACGAAAGCAGCTTCTCACTGTTCACTTTAGGAAGCAC<br>CACGATTCCAATTTCAAACCTACAGTTTATGAATGCCCTAAATGTGGTAAGGGCTATTTACGCTGGAGTAATATG<br>CATAAGCATGCTGAAAATTGTGGACTGGCGAGGGCAAAAGCTGTTGCATCCAGAAAAAGAAGCAAGGGCAAAAAG<br>AAAAAACGTGAGAACCTAAAGCATGTTAAGCAAGAAGTTGGCCCGGAATCCTTCCAAGATATCTGCACTGTGAAC<br>CATGAACGTTGTGCCAGTGAGATTGTTCCTGTTTTAGATGGAATAGAAGCAGGAGCTTCGAGCGAACAGAAAACA<br>GAAATGACATGTGAAATGCTTCTCAACATGATGGACAAATAA
		Deduced amino acid sequence	MQNVDLAERNGPFRGVGVVLSVQEGVCAFYDLELMQISILQEKAQAKNLESKSAERPLDMLLIELDRDKDVIALE<br>NKVQPLTPSEEGGEKELFSVREAKSRDCKASGDLLVSCNKYKEQEYVTPSEQAVTKNPKSQKSQKGKKAVFSCDL<br>CTFTSLRISSLNRHMKTHSDEKPHVCHLCLKAFRTATLLRNHVNAHTGTRPYKCSDCDMAFVTSGELARHRRYKH<br>TLEKPFKCSICKYSSVEASKLKRHIRSHTGERPYACYLCSYASKDTYKLKRHMITHSGEKPYECYVCQARFTQSG<br>TMKIHILQKHGENVPKYQCPHCNTFIARKSDLGVHLRNLHSYMAVAIKCSYCEAVFHERYALTQHKKTHKNEKRF<br>RCDQCSYACKQERHLIVHKRTHTGEKPFTCLCCSKSFQRKQLLTVHFRKHHDSNFKPTVYECPKCGKGYLRWSNM<br>HKHAENCGLARAKAVASRKRSKGKKKKRENLKHVKQEVGPESFQDICTVNHERCASEIVPVLDGIEAGASSEQKT<br>EMTCEMLLNMMDK

d

Species	Gene	Type	Sequence
Petromyzon marinus (sea lamprey)	*CTCF2*	Nucleotide coding sequence	GGGAGGCGCGGTGGAGTAGCGGGCAGGAGAGGAGGCGGTGGAGTAGGAAGGAACTCAGCCACTGCTGAGCGCAGT<br>CACCGCTGCCACCTCTGTGACCGGATCTTCGGCTCCGTCACGCTCCTACGCAACCACATCAACACCCACACCGGC<br>ACGAAGCCGCACCAGTGCCTCGAGTGTGCGATGGCGTTTGTAACCAAGGGGGAGTTGGTGCGGCACGGCCGCTAC<br>GTCCACACCGGCGAGAAGCCCTTCAAGTGCTCTGCATGCGAGTACACAAGTGTGGAGGTGAGCATGATGAAACGC<br>CACATGAGGTCGCATACTGGCGAGCGTCCGTTCCAGTGCAGCGCGTGCAGCTACGCTAGCAAGGACGCCTACAGG<br>CTCAAGCGGCACATGAGGACGCACTCCGGGGAGAAGCCGTACGAGTGCCCGGTGTGCCGGGCGCGGTTCACCCAG<br>AAGAGCTCCATGAAATCCCACGTGCAGCAGAAGCACACGGAGGAGGGGGCCACTCCACGCTTCTCTTGCCCGCAC<br>TGCGACGCTGTCACAGCACGCAAGAGTGACCTGGGCGTGCATCTGCGCAAGCAACATGCGCTGGTGCGTCGTGGC<br>CTGCGCTGCCGCTACTGCACGGCCACCTTCCACGAGCACTTTGCCCTGATGCAGCACCAGCGCTGCCACCACCAC<br>GAGAAGCACTTCAAGTGCGACCGCTGCCAGTACGCCTGTACACAGGAGCGACACATGATAGCTCACAAGCGATCT<br>CACACGGCCGGGAAGCCGTTCGACTCGGCCCAACTCCCCATCGACGATGAGGAGGCAAGGCGGGAGGAGGAGGAG<br>GAGGAAGAGGCGGTGGTGAAGGTGAAAGTCGAGGAAGAAGAGCAGAGTCAGGAGGAGGTGGAAGATTCCAAACCA<br>CCCTCTGCTAAGCGACGTCCCGCCCCGCCATCCAAAGCCAGACGAGGCCGACCGGCAAAGTCCAAAGAATAA
		Deduced amino acid sequence	GRRGGVAGRRGGGGVGRNSATAERSHRCHLCDRIFGSVTLLRNHINTHTGTKPHQCLECAMAFVTKGELVRHGRY<br>VHTGEKPFKCSACEYTSVEVSMMKRHMRSHTGERPFQCSACSYASKDAYRLKRHMRTHSGEKPYECPVCRARFTQ<br>KSSMKSHVQQKHTEEGATPRFSCPHCDAVTARKSDLGVHLRKQHALVRRGLRCRYCTATFHEHFALMQHQRCHHH<br>EKHFKCDRCQYACTQERHMIAHKRSHTAGKPFDSAQLPIDDEEARREEEEEEEAVVKVKVEEEEQSQEEVEDSKP<br>PSAKRRPAPPSKARRGRPAKSKE

References

1. Sadaie, M., Shinmyozu, K. & Nakayama, J. A conserved SET domain methyltransferase, Set11, modifies ribosomal protein Rpl12 in fission yeast. *J. Biol. Chem.* **283**, 7185-95 (2008).
2. Mehta, T.K. *et al.* Evidence for at least six Hox clusters in the Japanese lamprey (*Lethenteron japonicum*). *Proc. Natl. Acad. Sci. U. S. A.* **110**, 16044-9 (2013).
3. Simao, F.A., Waterhouse, R.M., Ioannidis, P., Kriventseva, E.V. & Zdobnov, E.M. BUSCO: assessing genome assembly and annotation completeness with single-copy orthologs. *Bioinformatics* **31**, 3210-2 (2015).
4. Altschul, S.F., Gish, W., Miller, W., Myers, E.W. & Lipman, D.J. Basic local alignment search tool. *J. Mol. Biol.* **215**, 403-10 (1990).
5. Stanke, M., Steinkamp, R., Waack, S. & Morgenstern, B. AUGUSTUS: a web server for gene finding in eukaryotes. *Nucleic Acids Res.* **32**, W309-12 (2004).
6. Kim, D. *et al.* TopHat2: accurate alignment of transcriptomes in the presence of insertions, deletions and gene fusions. *Genome Biol.* **14**, R36 (2013).
7. Langmead, B. & Salzberg, S.L. Fast gapped-read alignment with Bowtie 2. *Nat. Methods* **9**, 357-9 (2012).
8. Slater, G.S. & Birney, E. Automated generation of heuristics for biological sequence comparison. *BMC Bioinform.* **6**, 31 (2005).
9. Smith, J.J. *et al.* Sequencing of the sea lamprey (*Petromyzon marinus*) genome provides insights into vertebrate evolution. *Nat. Genet.* **45**, 415-21, 421e1-2 (2013).
10. Smit, A.F.A. & Hubley, R. RepeatModeler Open-1.0. [http://www.repeatmasker.org]. (2008-2015).
11. Smit, A.F.A., Hubley, R. & Green, P. RepeatMasker Open-4.0. [http://www.repeatmasker.org]. (2013-2015).
12. Trapnell, C. *et al.* Differential gene and transcript expression analysis of RNA-seq experiments with TopHat and Cufflinks. *Nat. Protoc.* **7**, 562-578 (2012).
13. Grabherr, M.G. *et al.* Full-length transcriptome assembly from RNA-Seq data without a reference genome. *Nat. Biotechnol.* **29**, 644-52 (2011).
14. Haas, B.J. *et al.* Improving the *Arabidopsis* genome annotation using maximal transcript alignment assemblies. *Nucleic Acids Res.* **31**, 5654-5666 (2003).
15. Haas, B.J. *et al. De novo* transcript sequence reconstruction from RNA-seq using the Trinity platform for reference generation and analysis. *Nat. Protoc.* **8**, 1494-1512 (2013).
16. Pearson, W.R. & Lipman, D.J. Improved tools for biological sequence comparison. *Proc. Natl. Acad. Sci. U. S. A.* **85**, 2444-8 (1988).
17. Roberts, A. & Pachter, L. Streaming fragment assignment for real-time analysis of sequencing experiments. *Nat. Methods* **10**, 71-73 (2013).
18. Langmead, B., Trapnell, C., Pop, M. & Salzberg, S.L. Ultrafast and memory-efficient alignment

of short DNA sequences to the human genome. *Genome Biol.* **10**, R25 (2009).

19. Zhang, Y. *et al.* Model-based analysis of ChIP-Seq (MACS). *Genome Biol.* **9**, R137 (2008).
20. Grant, C.E., Bailey, T.L. & Noble, W.S. FIMO: scanning for occurrences of a given motif. *Bioinformatics* **27**, 1017-8 (2011).
21. Lee, T.I., Johnstone, S.E. & Young, R.A. Chromatin immunoprecipitation and microarray-based analysis of protein location. *Nat. Protoc.* **1**, 729-48 (2006).
